# EBV-miR-BART1-5P activates AMPK/mTOR/HIF1 pathway via a PTEN independent manner to promote glycolysis and angiogenesis in nasopharyngeal carcinoma

**DOI:** 10.1371/journal.ppat.1007484

**Published:** 2018-12-17

**Authors:** Xiaoming Lyu, Jianguo Wang, Xia Guo, Gongfa Wu, Yang Jiao, Oluwasijibomi Damola Faleti, Pengfei Liu, Tielian Liu, Yufei Long, Tuotuo Chong, Xu Yang, Jing Huang, Mingliang He, Chi Man Tsang, Sai Wah Tsao, Qian Wang, Qiang Jiang, Xin Li

**Affiliations:** 1 Department of laboratory medicine, The Third Affiliated Hospital, Southern Medical University, Guangzhou, P.R. China; 2 Shenzhen Key Laboratory of Viral Oncology, the Clinical Innovation & Research Center (CIRC), Shenzhen Hospital, Southern Medical University, Shenzhen, China; 3 Department of Pathology, Zengcheng District People’s Hospital of Guangzhou City, Guangzhou, P.R. China; 4 Department of Biomedical Sciences, City University of Hong Kong, Hong Kong SAR, China; 5 School of Biomedical Sciences, Li Ka Shing Faculty of Medicine, University of Hong Kong, Hong Kong SAR, China; 6 Department of Anatomical and Cellular Pathology, State Key Laboratory of Translational Oncology, Faculty of Medicine, Chinese University of Hong Kong, Hong Kong SAR, China; 7 Zhujiang Hospital, Southern Medical University, Guangzhou, P.R. China; 8 Department of Oncology, Henan Provincial People’s Hospital, Zhengzhou, P.R. China; Tulane Health Sciences Center, UNITED STATES

## Abstract

Abnormal metabolism and uncontrolled angiogenesis are two important characteristics of malignant tumors. The occurrence of both events involves many key molecular changes including miRNA. However, EBV encoded miRNAs are rarely mentioned as capable of regulating tumor metabolism and tumor angiogenesis. Here, we reported that one of the key miRNAs encoded by EBV, EBV-miR-Bart1-5P, can significantly promote nasopharyngeal carcinoma (NPC) cell glycolysis and induces angiogenesis in vitro and in vivo. Mechanistically, EBV-miR-Bart1-5P directly targets the α1 catalytic subunit of AMP-activated protein kinase (AMPKα1) and consequently regulates the AMPK/mTOR/HIF1 pathway which impelled NPC cell anomalous aerobic glycolysis and angiogenesis, ultimately leads to uncontrolled growth of NPC. Our findings provide new insights into metabolism and angiogenesis of NPC and new opportunities for the development of targeted NPC therapy in the future.

## Author summary

The Epstein-Barr virus (EBV), the first reported human tumor virus found to encode miRNAs, which closely related to malignant progression of tumors. In our study, we have observed that EBV-miR-BART1-5P, an EBV-BARTs encoded miRNA, promotes glycolysis and induces angiogenesis in NPC. Interestingly, we showed that overexpression of EBV-miR -BART1-5P and restored PTEN at the same time, did not completely reverse the phenotypes of glycolysis, angiogenesis and proliferation, suggesting that EBV-miR-BART1-5P can mediate glycolysis and induction angiogenesis by a PTEN-independent manner. Further mechanism exploration demonstrated that EBV-miR-BART1-5P has important roles in cancer cell glucose metabolism and angiogenesis by inhibiting AMPKα1 and PTEN, which provides a molecular basis for the regulation of AMPK/mTOR/HIF1 and PTEN/FAK, Shc, AKT pathways, respectively.

## Introduction

The development of malignant tumors is divided into several stages: malignant transformation of cells, clonal proliferation of transformed cells, local infiltration and distant metastasis. An abnormal supply of energy and sustained angiogenesis, two of the ten characteristics of tumor development [1], maintain the growth during the different stages of the cancer. They play an important role in cancer progression, including regulation of cancer growth, invasion and metastasis [2].

Cancer cells have a unique energy metabolism phenotype that consumes more glucose and converts pyruvate to lactate, even under normoxia, which is called the Warburg effect or aerobic glycolysis [3]. Aerobic glycolysis gives cancer cells a growth advantage not only by providing more glycolytic intermediates for various biosynthetic pathways, but also by minimizing the production of reactive oxygen species in the mitochondria [4,5]. Due to the rapid proliferation of cancer cells, hypoxia occurs to which cancer cells adapt by upregulating their glycolysis. This also leads to an increased acid production, which leads to a significant decrease in the local extracellular pH. The microenvironment acidification promotes cancer invasion and angiogenesis by disrupting adjacent normal cells and by acid-induced extracellular matrix (ECM) degradation [6–8]. However, in a variety of tumors, including nasopharyngeal carcinoma (NPC), the molecular mechanism leading to abnormal aerobic glycolysis remains obscure.

MiRNAs are highly conserved noncoding RNAs that regulate a variety of biological processes [9]. In recent studies, multiple cellular miRNAs, such as miR-199a-5p[10], miR-143 [11–15], miR-451 [16], miR-210 [17], miR-29b [18], miR-195-5p [19], miR-375 [20,21] have been reported to participate in the energy metabolism process by regulating the gene expression of metabolic-associated genes through posttranscriptional repression and mRNA degradation [22]. It is known that virus-encoded miRNAs can also regulate cell energy metabolism and angiogenesis[23–26], but the underlying mechanism is still largely unknown.

The Epstein-Barr virus (EBV), which is aetiologically linked to several cancers including Hodgkin lymphoma, Burkitt’s lymphoma, gastric cancer and NPC [27], is the first reported human tumor virus found to encode miRNAs [28]. Until now, EBV encodes 48 mature miRNAs that have been identified within two regions of the EBV genome. The BamHI fragment H rightward reading frame 1 (BHRF1) gene generating four mature miRNAs and the BamHI fragment A rightward transcript (BART) region producing 44 mature miRNAs. BART-miRNAs are highly expressed in epithelial malignancies including NPC and EBV-associated gastric cancers [29]. At present, EBV-miR-BARTs have become more and more important in the development of NPC where they have been reported to participate in a series of pathological processes such as proliferation, apoptosis, invasion and metastasis [30]. In a previous study, we have demonstrated that EBV-miR-BART1-5P and EBV-miR-BART1-3P directly target the PTEN-AKT signaling pathway to mediate NPC cells metastasis [31]. However, few articles have linked EBV-miR-BARTs to aerobic glycolysis and angiogenesis.

In this study, we have observed that EBV-miR-BART1-5P promotes glycolysis and induces angiogenesis in NPC. The underlying molecular mechanism revealed to directly target the α1 catalytic subunit of AMP-activated protein kinase (AMPKα1) and consequently regulates the AMPK/mTOR/HIF1 pathway which ultimately leads to growth of NPC cells.

## Results

### EBV-miR-BART1-5P enhances glycolysis of NPC cells

In aerobic glycolysis, glucose is the starting material, while lactate is the final product. To validate the roles of BART1 in NPC glycometabolism, we detected the secretion of lactate and the consumption of glucose by transiently transfecting BART1-3P and BART1-5P mimics into 7 cell lines (2 EBV-negative NPC cell lines and 5 EBV-negative epithelial cell lines) respectively. Compared with BART1-3P, BART1-5P significantly increased the production of lactate and the consumption of glucose in all 7 cell lines (**S1 Fig**). This suggests that BART1-5P affects more genes related to glucose metabolism.

**Fig 1:**
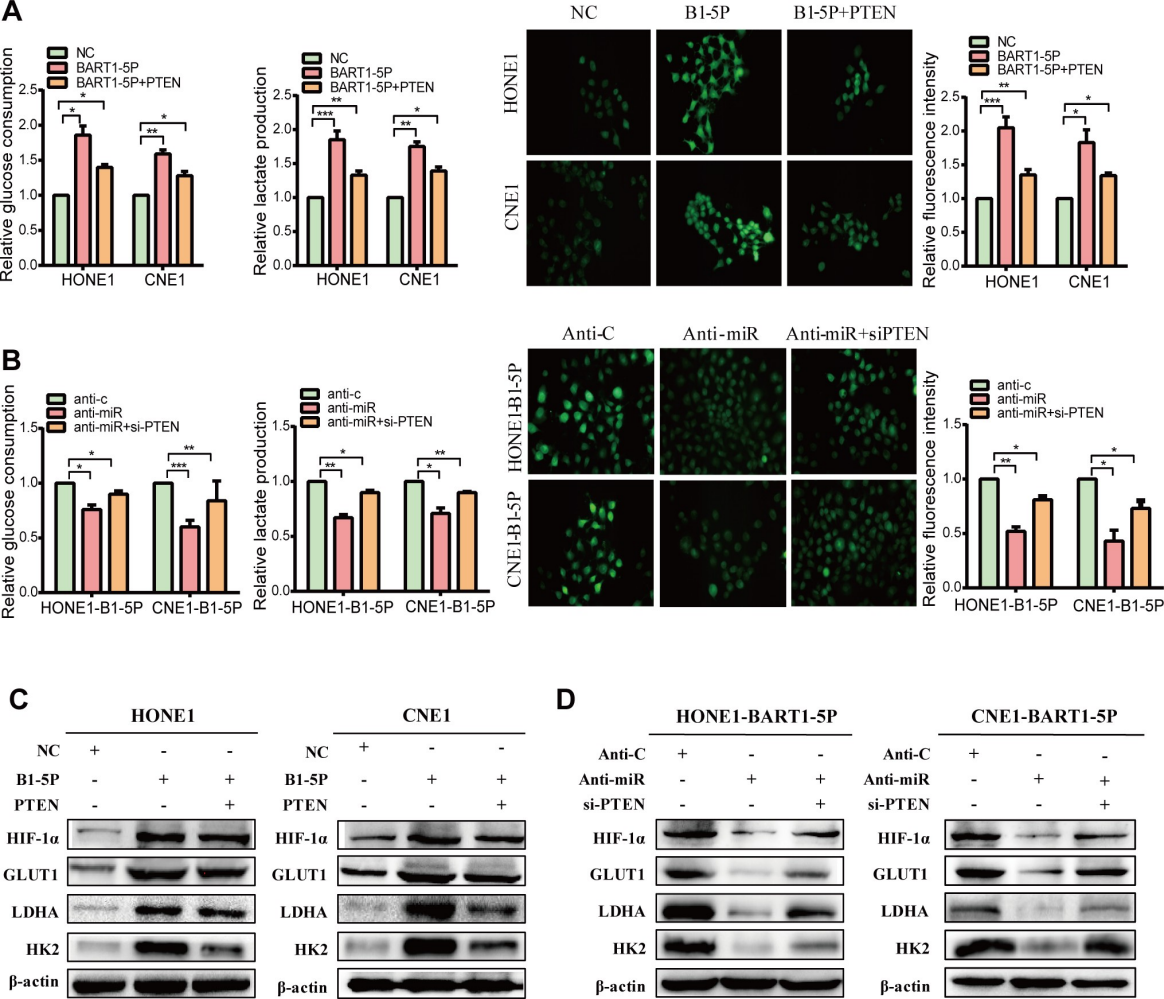
EBV-miR-BART1-5P enhances glycolysis of NPC cells. (**A**) Lactate production, glucose consumption and 2-NBDG uptake (Magnification, ×100) in HONE and CNE1 cells after transfection EBV-miR-BART1-5P mimic alone or co-transfection EBV-miR-BART1-5P mimic and PTEN plasmid. (**B**) Lactate production, glucose consumption and 2-NBDG uptake in HONE-BART1-5P and CNE1-BART1-5P cells after transfection anti-miR alone or co-transfection anti-miR and si-PTEN. Anti-Control abbreviated anti-c. The data were shown as the mean ± s.e.m. (*P<0.05, **P<0.01 and ***P<0.001). (**C**) The glucose metabolism associated proteins were detected by western blot in HONE and CNE1 cells after the introduction of EBV-miR-BART1-5P or co-transfection EBV-miR-BART1-5P mimic and PTEN plasmid. (**D**) GLUT1, HK2, LDHA and HIF-1α were detected by western blot in HONE-BART1-5P and CNE1-BART1-5P cells after the introduction of anti-miR alone or co-transfection anti-miR and si-PTEN.

To further determine the role of BART1-5P on NPC glycometabolism we used lentiviral particles carrying BART1-5P precursor to generate two EBV-negative cell lines (One is NPC Cell line, another is an EBV-negative epithelial cell line.) stably expressing BART1-5P (HONE-BART1-5P and CNE1-BART1-5P). The expression levels of BART1-5P in these two cell lines were within a similar physiological range to pooled NPC tissue samples **(S2 Fig)**. NPC cells with exogenous expression of BART1-5P secreted more lactate **(Fig 1A)**, consumed more glucose **(Fig 1A)** and exhibited more cellular ATP levels **(S3A Fig)**. Moreover, expression of BART1-5P significantly increased recipient cells' uptake of 2-NBDG, a fluorescent analogue of glucose which has been used to assess glucose transport in various cell types [32,33] **(Fig 1A)**. On the contrary, silencing of BART1-5P reversed the changes in the secretion of lactate, consumption of glucose, uptake of glucose **(Fig 1B)** and ATP levels **(S3B Fig).** GLUT1, HK2 and LDHA are pivotal enzymes in glucose metabolism catalyzing many key steps in the glycolytic pathway. Hypoxia-inducible factor (HIF)-1α has been identified as a contributor to aerobic glycolysis by regulating of expression of various glycometabolism-associated genes [34]. We observed that BART1-5P significantly increased the expression of GLUT1, HK2, LDHA and HIF-1α **(Fig 1C).** Furthermore, downregulation of BART1-5P reduced the GLUT1, HK2, LDHA and HIF-1α levels **(Fig 1D).** Interestingly, overexpression of PTEN did not completely attenuate the effect of BART1-5P on glucose metabolism **(Fig 1A–1D)**, suggesting that PTEN is not an independent factor affecting glucose metabolism.

The above results collectively indicate that BART1-5P enhances glycolysis in NPC cells.

### EBV-miR-BART1-5P induces angiogenesis in vitro and vivo

**Fig 2:**
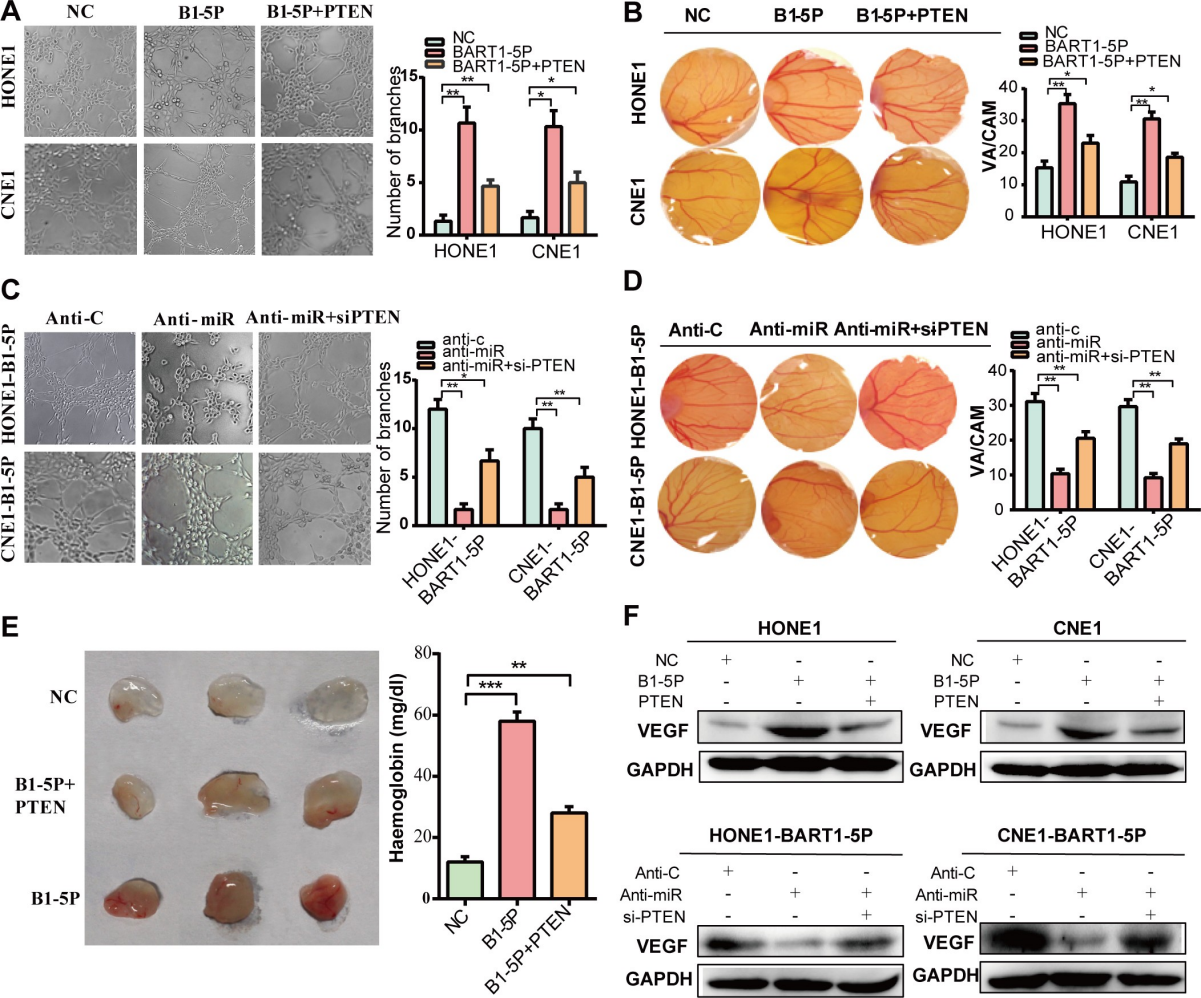
EBV-miR-BART1-5P induces angiogenesis in vitro and vivo. (**A, C**) Tube formation potential of HUVEC cells following the exposure to conditioned media collected from NPC cells treated with EBV-miR-BART1-5P or anti-miR. (**B, D**) CAM angiogenesis was performed with NPC cells overexpressing or inhibiting EBV-miR-BART1-5P. Representative images of new blood vessel formation are shown(left), new blood vessels were counted under a dissecting microscope(right). VA = Vascular area CAM = Chorioallantoic membrane area (mm^2^). (**E**) Representative images (left) and haemoglobin quantification (right) of the in vivo Matrigel plug assay. The conditioned media collected from HONE1 cells transfected EBV-miR-BART1-5P alone or co-transfected EBV-miR-BART1-5P and PTEN plasmid were mixed with Matrigel. (**F**) VEGF was detected by western blot in BART1-5P/anti-miR treated NPC cells. Restoring the expression of PTEN in BART1-5P/anti-miR treated NPC cells only partially influenced the phenotype of angiogenesis. The data were shown as the mean ± s.e.m. (*P<0.05, **P<0.01 and ***P<0.001).

In the majority of solid tumors, as the tumor grows, it becomes more and more difficult for the inner cancer cells to obtain sufficient oxygen from the blood. As a result, HIF-1α, a subunit of the heterodimeric transcription factor HIF-1, is overexpressed in the hypoxic microenvironment of most human cancers. HIF-1α regulates vascular endothelial growth factor (VEGF) [35], the principal mediator of angiogenesis in a number of cancers, under normal physiologic conditions. In the present study, we found that BART1-5P promotes the expression of HIF-1α. To directly detect whether BART1-5P promotes angiogenesis, the chorioallantoic membrane (CAM) assay was used. We stably expressed BART1-5P in HONE1, CNE1 and HK1 cells and applied the cell supernatant over chorioallantoic membranes via sponges [36]. BART1-5P, but not NC, significantly promoted neovascularization **(Fig 2B and 2D and S4A Fig)**. In contrast, silencing BART1-5P significantly reduced angiogenesis **(Fig 2B and 2D and S4B Fig)**. A similar result was seen in the endothelial tube formation assay. Compared with the NC, upregulation of BART1-5P significantly increased the tube formation potential. In agreement with the upregulation results, downregulation of BART1-5P reduced the tube formation potential **(Fig 2A and 2C).** Next, an in vivo matrigel plug assay was performed using conditioned media collected from BART1-5P or NC NPC cells. The results showed that BART1-5P overexpression led to a substantial increase in haemoglobin **(Fig 2E)**, a surrogate marker for functional blood flow [37]. Earlier it was shown that PTEN could also inhibit angiogenesis by regulating the expression of VEGF expression through AKT activation [38]. At the protein level, BART1-5P significantly promoted the expression of VEGF, whereas restoring the expression of PTEN in BART1-5P/anti-miR treated NPC cells only partially influenced the expression of VEGF **(Fig 2F)**. A similar phenomenon can be observed in the CAM **(Fig 2B and 2D and S4 Fig)**, endothelial tube formation **(Fig 2A and 2C)**.and matrigel plug assay **(Fig 2E)**, suggesting that BART1-5P affects multiple genes including PTEN to promote angiogenesis in NPC.

### EBV-miR-BART1-5P promotes the proliferation of NPC cells

**Fig 3:**
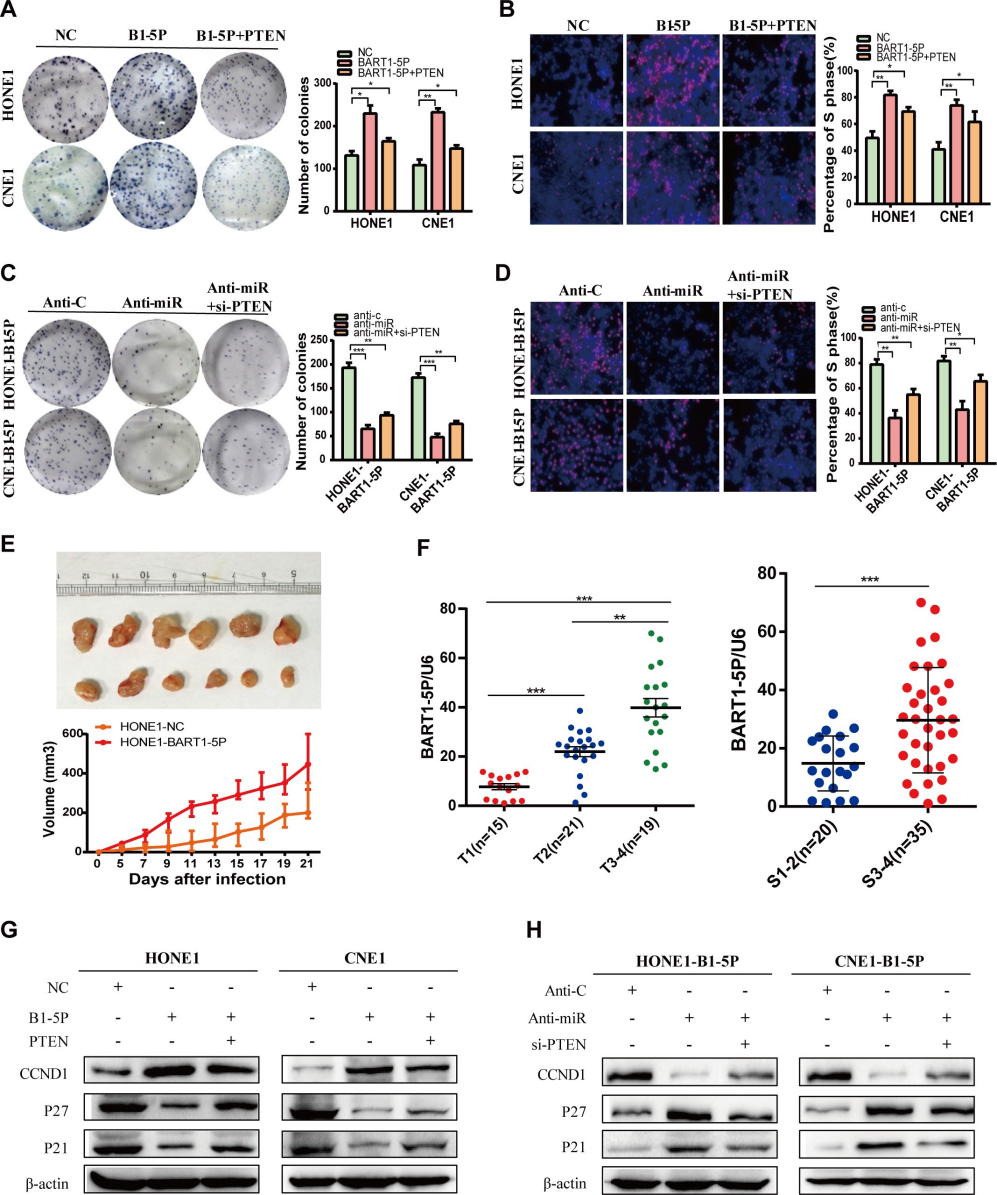
EBV-miR-BART1-5P promotes the proliferation of NPC cells. (**A, C**) colony formation assay, (**B, D**) EdU incorporation assays of NPC cells performed after transfection with NC, anti-c, EBV-miR-BART1-5P mimics, inhibitor and/or PTEN plasmid, si-PTEN as indicated. The data were shown as the mean ± s.e.m. (*P<0.05, **P<0.01 and ***P<0.001). (**E**) The in vivo effect of EBV-miR-BART1-5P was evaluated in xenograft mouse models bearing tumours originating from HONE1 cells, n = 6/group. Tumour volume was periodically measured for each mouse and tumour growth curves was plotted. Parametric generalized linear model with random effects. (**F**) EBV-miR-BART1-5P expression normalized to U6 snRNA was detected by qPCR in NPC samples with clinical stage and T stage. The data were shown as the mean ± s.e.m. (*P<0.05, **P<0.01 and ***P<0.001). (**G, H**) Expression of CCND1, P27 and P21 were detected after transfection of EBV-miR-BART1-5P mimic/inhibitor or PTEN plasmid/si-PTEN. β-actin was used as a loading control.

Regarding energy metabolism, BART1-5P promotes potential NPC cell proliferation. Hence, we examined the effect of BART1-5P expression on the growth of NPC cells in vivo and in vitro. Using colony formation **(Fig 3A and 3C)**, Edu incorporation assays **(Fig 3B and 3D)** and cell cycle analysis **(S5A and S5B Fig)**, we observed that BART1-5P significantly promoted cell growth and G1/S transition in NPC cells. Conversely, silencing BART1-5P significantly inhibited cell proliferation and mediated G1/S transition in NPC cells **(Fig 3A–3D)**. We also found that BART1-5P overexpression enhanced CCND1 expression but downregulated the expression of p27 and p21. BART1-5P inhibitors rescued these effects **(Fig 3G and 3H)**.

Furthermore, HONE1-BART1-5P cells and HONE1-NC cells were subcutaneously injected into nude mice. Upregulation of BART1-5P significantly increased tumor growth when compared with NC **(Fig 3E)**. To further confirm that BART1-5P can increase tumor growth, we inject nude mice with HONE-EBV-miR-ctrl and HONE-EBV-BART1-5P-antago-miR (Purchased from Shanghai GenePharma Co., Ltd) cells to evaluate the size of tumor decline. Experimental results showed that antago-mir-BART1-5P decreased tumor growth when compared with antagomir-ctrl **(S7 Fig).**

In 55 NPC tissues, we observed that the expression of BART1-5P was positively correlated with the T stage **(Fig 3F)** and clinical stage of NPC **(Fig 3F)**, indicating a correlation between BART1-5P and the growth of NPC. Restoring PTEN expression in these experiments yielded similar results as in the glycolysis and angiogenesis experiments **(Fig 3)**. These results suggest that BART1-5P promotes the growth of NPC cells.

### EBV-miR-BART1-5P directly targets cellular AMPKα1 gene

**Fig 4:**
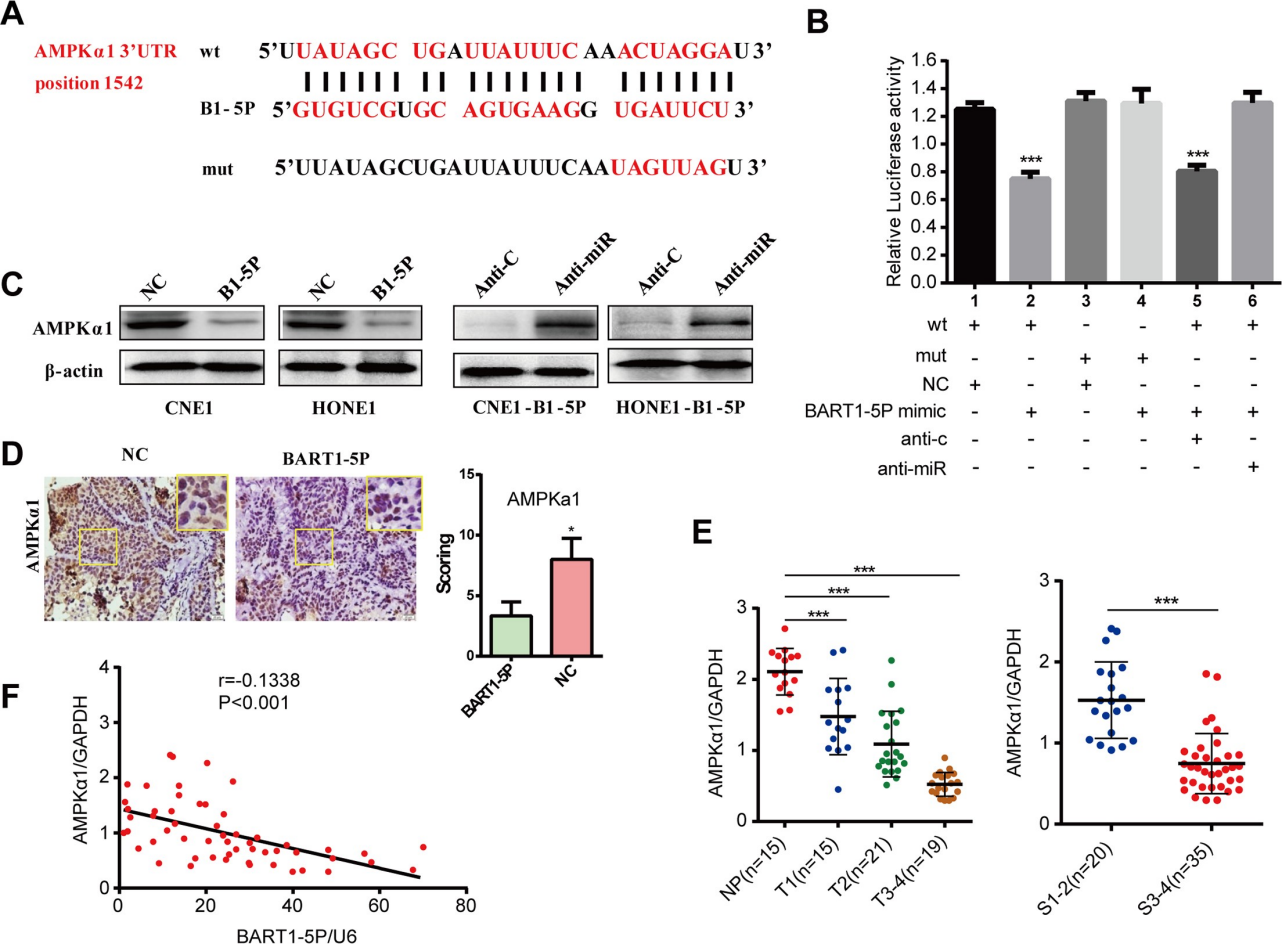
EBV-miR-BART1-5P directly targets cellular AMPKα1 gene. (**A**) EBV-miR-BART1-5P and its putative binding sequences in the 3’UTR of AMPKα1. Mutation was generated in the complementary site that binds to the seed region of BV-miR-BART1-5P. (**B**) HEK293T cells were cotransfected with EBV-miR-BART1-5P mimic or NC and luciferase reporter carrying either the predicted miRNA target site in AMPKα1 3’UTR (wt) or its corresponding mutant (mut). Inhibitor of EBV-miR-BART1-5P by anti-miR and anti-control (anti-c) was also performed. The data were shown as the mean ± s.e.m. (***P<0.001). (**C**) Western blotting analysis of endogenous AMPKα1 protein expression levels in CNE1 and HONE1 cells treated with EBV-miR-BART1-5P mimic or inhibitor. β-actin was used as a loading control. (**D**) AMPKα1 expression was detected using immunohistochemistry assay in tumour tissues derived from tumorigenesis in nude mice models. Magnification, ×400. (**E**) The levels of AMPKα1 was evaluated by qPCR in NPC and NP tissue specimens (with clinical stage and T stage). Normalized to GAPDH. (**F**) The correlation between EBV-miR-BART1-5P and AMPKα1 expression levels was calculated. The data were shown as the mean ± s.e.m. (*P<0.05, **P<0.01 and ***P<0.001).

To investigate glucose metabolism and angiogenesis-related BART1-5P-target genes we performed deep sequencing. Predicted EBV-miR-BART1-5P target genes were retrieved from TargetScan and RNAhybird. One of the potential candidates AMPKα1 is an important energy sensor that regulates the cellular metabolism to maintain energy homeostasis and affects angiogenesis by activating the mTOR pathway. Bioinformatics analysis showed that the 3'UTR of AMPKα1 was well matched with the seed sequence of EBV-miR-BART1-5P **(Fig 4A)**.

To clarify whether BART1-5P could directly target AMPKα1, we performed a luciferase reporter assay. BART1-5P significantly reduced the luciferase activity of the wt AMPKα1 3'UTR reporter vector and this effect was abolished when the AMPKα1 3'UTR binding site was mutated. In addition, anti-miR increased the luciferase activity of the wt AMPKα1 3'UTR reporter vector rather than of the mt 3'UTR **(Fig 4B)**.

We detected the effects of BART1-5P on AMPKα1 mRNA and protein expression in NPC cell lines, xenografted tumors and tissue samples. The expression of AMPKα1 was significantly lower in both HONE-BART1-5P and CNE1-BART1-5P cell lines and in xenografted tumors when compared with the relative controls as revealed by western blotting **(Fig 4C)** and immunohistochemical (IHC) staining **(Fig 4D)**. We detected AMPKα1 expression in 55 NPC and 15 NP tissue samples. The results showed that the downregulation of AMPKα1 in clinical tumor samples was associated with the T stages and advanced clinical stage of NPC **(Fig 4E)**. Moreover, the expression of AMPKα1 was negatively associated with BART1-5P expression **(Fig 4F)**. we also collected 20 new nasopharyngeal carcinoma (NPC) samples and 10 chronic nasopharyngitis (NP) samples to be used for immunohistochemical staining. The result showed the downregulation of AMPKα1 in NPC samples compared with the NP samples **(S6 Fig)**.

Collectively, these results indicate that AMPKα1 is a direct cellular target of EBV-miR-BART1-5P in NPC.

### EBV-miR-BART1-5P regulates the AMPK/mTOR/HIF1 signaling pathways

**Fig 5:**
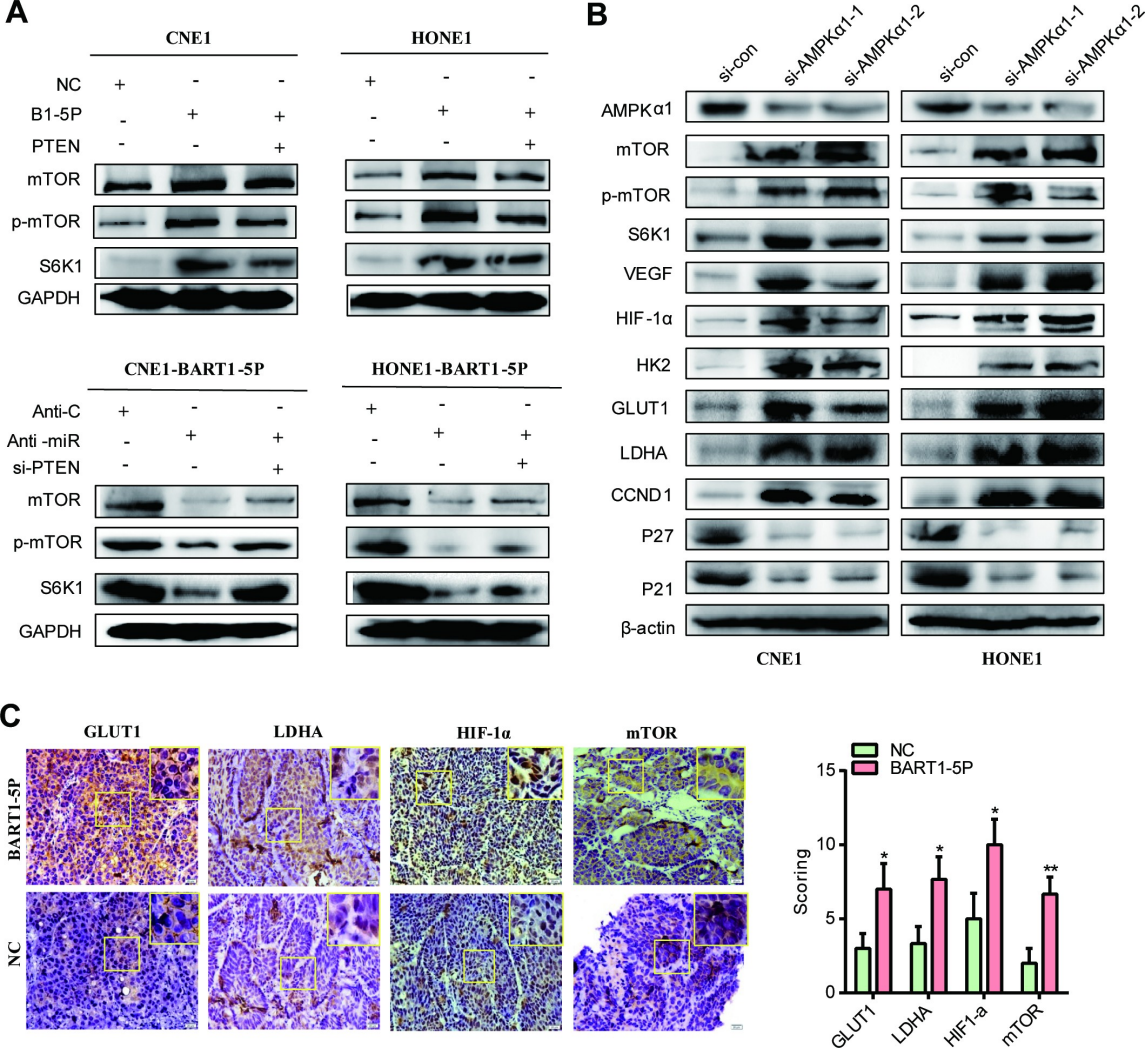
EBV-miR-BART1-5P regulates the AMPK/mTOR/HIF1 signaling pathways. (**A**) mTOR, p-mTOR and S6K1 were detected by western blot in CNE1 and HONE1 cells transfected with EBV-miR-BART1-5P mimic or co-transfected EBV-miR-BART1-5P mimic and PTEN plasmid. HONE-BART1-5P and CNE1-BART1-5P cells treated with anti-miR or anti-miR and si-PTEN were also performed. GAPDH was used as a loading control. (**B**) Western blot of endogenous AMPKα1, mTOR, p- mTOR, S6K1, VEGF, HIF-1α, GLUT1, LDHA, CCND1, P27 and p21 protein expression levels in HONE1 and CNE1 cells treated with si-AMPKα1 or si-control. β-actin was used as a loading control. (**C**) GLUT1, LDHA and HIF-1α expression was detected using immunohistochemistry assay in tumour tissues derived from tumorigenesis in nude mice models. Magnification, ×400.

We examined alterations in the expression of key components of the AMPKα1 pathway in NPC [39,40]. We observed that the upregulation of BART1-5P significantly reduced the protein expression of AMPKα1 but increased the level of mTOR, p-mTOR and S6K1, whereas silencing of BART1-5P attenuated mTOR, p-mTOR and S6K1 levels when compared with the relative control **(Fig 5A)**. Further, we found that the protein levels of mTOR, p-mTOR, S6K1, VEGF, HIF-1α, HK2, GLUT1, LDHA and CCND1 were significantly increased, while AMPKα1, p21 and p27 were reduced after AMPKα1 siRNA treatment **(Fig 5B)**. Restoring PTEN expression in NPC cells could only partially restore the expression of mTOR and downstream proteins **(Fig 5A)**. IHC staining of sections of tumor xenografts showed that upregulation of BART1-5P significantly reduced the expression of AMPKα1 but increased HIF-1α, GLUT1, LDHA and mTOR when compared with the relative control **(Fig 5C)**. These data indicate that EBV-miR-BART1-5P activates the AMPK/mTOR/HIF1 signaling pathway.

### Restoration of AMPKα1 rescued the phenotypes generated by EBV-miR-BART1-5P

**Fig 6:**
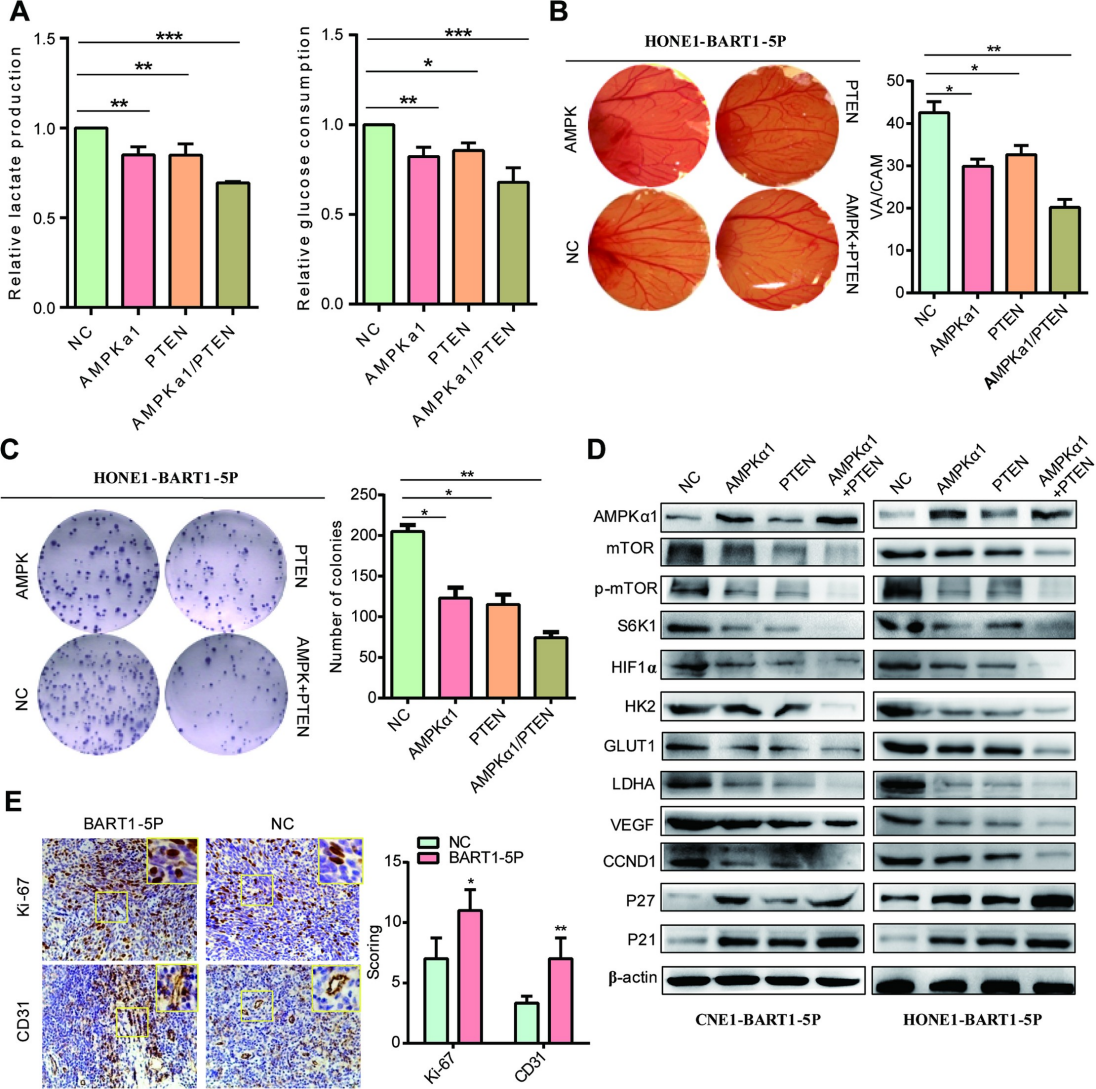
Restoration of AMPKα1 rescued the phenotypes generated by EBV-miR-BART1-5P. (**A**) Lactate production and glucose consumption, (**B**) CAM angiogenesis, (**C**) colony formation detected in HONE-BART1-5P cells after transfection AMPKα1 plasmid, PTEN plasmid and both of them. The data were shown as the mean ± s.e.m. (*P<0.05, **P<0.01 and ***P<0.001). (**D**) AMPKα1, mTOR, p- mTOR, S6K1, VEGF, HIF-1α, GLUT1, LDHA, CCND1, P27 and p21 protein expression levels in HONE-BART1-5P cells treated with AMPKα1 plasmid, PTEN plasmid and both of them. β-actin was used as a loading control. (**E**) Ki-67 and CD31 expression was detected using immunohistochemistry assay in tumour tissues derived from tumorigenesis in nude mice models. Magnification, ×400.

We transfected HONE-BART1-5P cells with the AMPKα1 expression vector, the PTEN expression vector or both. Restoration of either AMPKα1 or PTEN expression significantly reduced the secretion of lactate, consumption of glucose **(Fig 6A)**, angiogenesis **(Fig 6B and 6E)** and proliferation **(Fig 6C and 6E)** of NPC cells when compared with the negative vector control. Western blotting showed that the reconstitution of AMPKα1 and PTEN decreased the level of mTOR, p-mTOR, S6K1, VEGF, HIF-1α, HK2, GLUT1, LDHA and CCND1 but increased p21 and p27 when compared with NC **(Fig 6D).** As a result, restoration of AMPKα1 and PTEN reduced glycolysis, angiogenesis and proliferation of NPC cells.

Moreover, we detected related proteins in AMPK/mTOR/HIF pathway after treatment with aminoimidazole-4-carboxamide (AICAR, an activator of AMPK, purchased from Sigma-Aldrich, catalog number A9978, 100μM) and AMPK inhibitor Dorsomorphin. As expected, the experimental results of AICAR treatment exhibit AMPK/mTOR/HIF pathway activating effect as similarly to AMPKα1 rescue in stably expressing BART1-5P cell lines **(S8 and S9 Figs)**. we also tested the key proteins in the AMPK/mTOR/HIF pathway after treatment with AMPK inhibitor Dorsomorphin (10μM, Sigma-Aldrich, catalog number P5499). Experimental results of AICAR treatment exhibit AMPK/mTOR/HIF pathway inhibiting effect as similar to si-AMPKα1 in HONE1-EBV cells **(S10 and S11 Figs)**.

### Silencing of endogenous EBV-miR-BART1-5P attenuates the phenotypes in EBV-positive NPC cells

We quantified the expression of EBV-miR-BART1-5P in three EBV-positive NPC cell lines (C666-1, HONE1-EBV, and HK1-EBV) and compared it with NPC clinical samples by qRT-PCR **(S13 Fig)**. We used the HONE1-EBV cell line as a representative to further validate the role of BART1-5P in NPC glucose metabolism, angiogenesis and to clarify the molecular mechanisms.

First, we detected the expression of HIF-1α, GLUT1, LDHA and the related proteins of AMPK/mTOR/HIF pathway within HONE1-EBV and HONE1 cell lines by western blotting assay. The result was displayed in the supplementary files **(S12A Fig)**. Further, BART1-5P significantly increased the expression of HIF-1α, GLUT1, LDHA and the related proteins of AMPK/mTOR/HIF pathway in normal epithelium (NP460 cells) cells **(S12B Fig)**.

**Fig 7:**
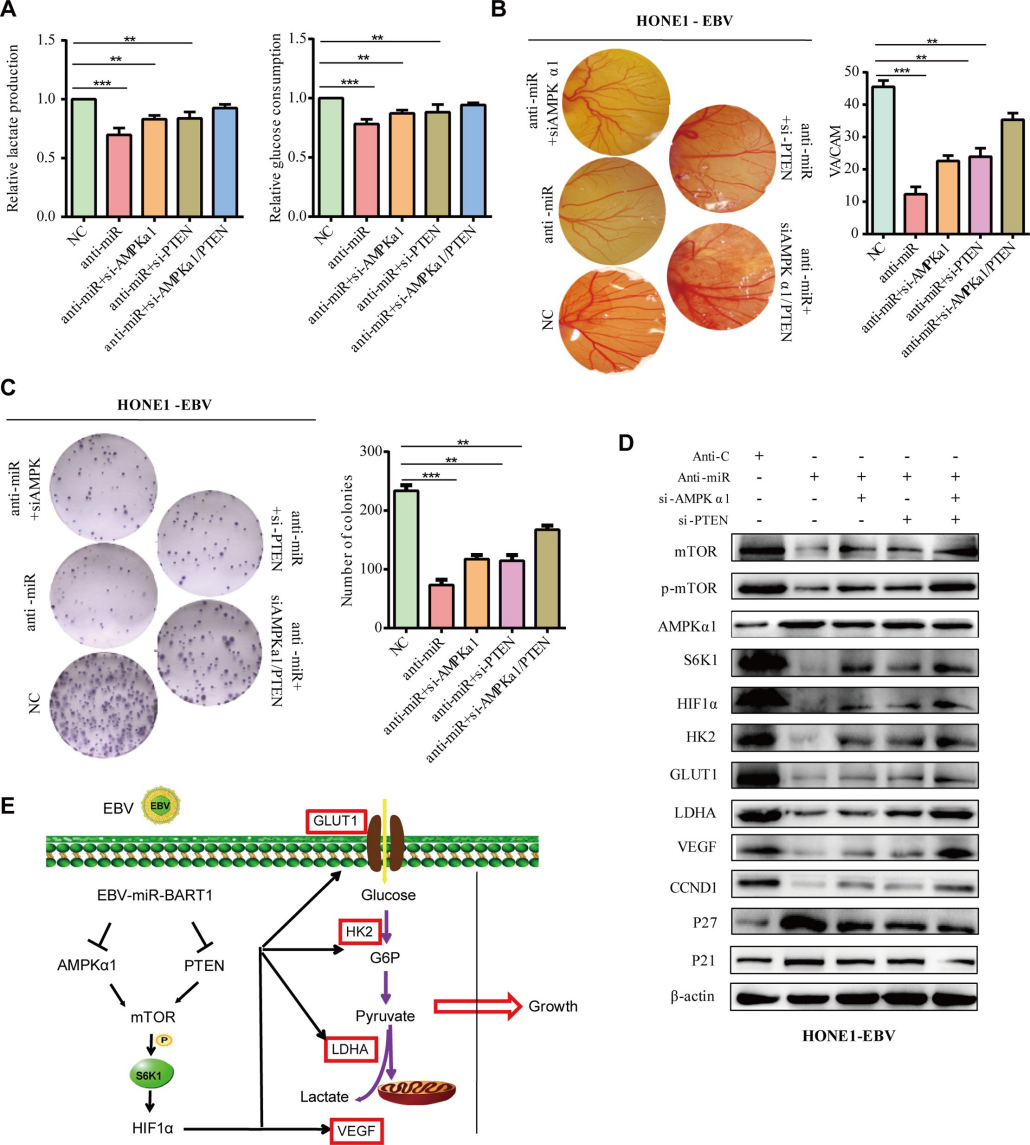
Silencing of endogenous EBV-miR-BART1-5P attenuates the phenotypes in EBV-positive NPC cells. (**A**) Lactate production and glucose consumption, (**B**) CAM angiogenesis, (**C**) colony formation detected in HONE-EBV cells after transfection anti-control, anti-miR, anti-miR+si-AMPKα1, anti-miR+si-PTEN and anti-miR+si-AMPKα1+si-PTEN, respectively. The data were shown as the mean ± s.e.m. (*P<0.05, **P<0.01 and ***P<0.001). (**D**) AMPKα1, mTOR, p- mTOR, S6K1, VEGF, HIF-1α, GLUT1, LDHA, CCND1, P27 and p21 protein expression levels in HONE-EBV cells treated with anti-control, anti-miR, anti-miR+si-AMPKα1, anti-miR+si-PTEN and anti-miR+si-AMPKα1+si-PTEN, respectively. β-actin was used as a loading control. (**E**) A proposed model demonstrating the role of EBV-miR-BART1-5P in the glycolysis, angiogenesis and proliferation of NPC cells.

HONE-EBV cells were transfected with anti-miR, anti-miR and si-AMPKα1, anti-miR and si-PTEN or with all three, respectively. The secretion of lactate, consumption of glucose **(Fig 7A)**, angiogenesis **(Fig 7B)** and proliferation **(Fig 7C)** of NPC cells gradually increased when compared with NC. An additive effect was obtained when both si-AMPKα1 and si-PTEN were co-transfected. Similarly, western blotting demonstrated that downregulation of endogenous BART1-5P increased AMPKα1, P21 and P27 expression but reduced mTOR, p-mTOR, S6K1, VEGF, HIF-1α, HK2, GLUT1, LDHA and CCND1 expression when compared with the anti-control **(Fig 7D)**. Thus, these data validate that BART1-5P promotes glycolysis, angiogenesis and proliferation of NPC cells by regulating the AMPK/mTOR/HIF1 signaling pathway.

## Discussion

Tumor cells need to adjust their energy metabolism for cell growth and division as rapid growth of solid tumors can lead to tissue hypoxia. By switching to glycolysis, tumor cells successfully adapted to local hypoxia. Furthermore, acidification of the microenvironment due to an increased lactate concentration can promote tumor proliferation [41], invasion and angiogenesis [42,43]. The key regulator of the glycolytic response is the transcription factor HIF-1α, which activates a number of pivotal enzyme in glucose metabolism and catalyzes the irreversible rate-limiting step and also activates VEGF to promote angiogenesis [4]. In fact, there is growing evidence that the ‘glycolytic switch’ occurs before the ‘angiogenic switch’ [44], and that increased glucose uptake is observed to coincide with the transition from pre-malignant lesions to invasive cancer [45,46]. This further suggests that the glycolytic phenotype plays a vital role in the development and growth of tumors.

NPC is typically characterized by rapid progress and high metastatic potential [47]. Investigating the key mechanisms in the development of NPC contributes to a thorough understanding of the molecular mechanisms underlying the development of tumors and to develop new clinical management strategies. Previously, we observed that EBV-miR-BART1 is highly expressed in NPC and is related to patients with advanced stages. Further, EBV-miR-BART1 promotes NPC cells invasion and metastasis by directly targeting PTEN [31]. At present, we found that EBV-miR-BART1-5P activates the AMPK/mTOR/HIF1 pathway by targeting AMPKα1 to upregulate the glycolysis of NPC cells, to induce angiogenesis, and ultimately to promote the growth of NPC cells.

EBV-miRNA-BARTs regulate both viral and cellular genes in NPC cells. Such as, EBV-miR-BART3* promotes the growth and transformation of NPC cells by incompletely matching with the Dice1 gene [48]. EBV-miR-BART9 promotes tumor cell invasion by targeting E-cadherin, while targeting PTEN promotes tumor cell proliferation [49]. EBV-miR-BART5 targets PUMA to protect NPC cells from apoptosis [50]. EBV-miR-BART7-3p promotes an EMT phenotype by targeting PTEN, eventually leading to NPC metastasis [51]. Previously, we observed that large numbers of metabolically related genes were abnormally expressed when EBV-miR-BART1 was overexpressed in NPC cells, suggesting that EBV-miR-BART1 may contribute to NPC energy metabolism [52]. Pre-experimental results suggested that EBV-miRNA-BART1-5P, but not EBV-miRNA-BART-3P, plays a critical role in glycolysis in NPC cells. To verify this hypothesis, we regulated the expression of EBV-miRNA-BART1-5P in both EBV-negative and EBV-positive NPC cells and found that EBV-miRNA-BART1-5P significantly promotes glycolysis and induces angiogenesis of NPC cells.

The target gene and related signaling pathway of EBV-miR-BART1-5P were found by RNA-deep sequencing, bioinformatics prediction, literature search and luciferase reporter assay. We demonstrate for the first time that metabolic sensor AMPKα1 is the critical cellular target of EBV-miR-BART1-5P in NPC. Exogenous EBV-miR-BART1-5P expression can attenuate the expression of endogenous AMPKα1, and AMPKα1 re-expression was able to reverse the EBV-miR-BART1-5P-mediated phenotypes.

AMPK is the key energy sensor for the body and the main regulator of cellular and organic energy stabilization. It coordinates a variety of metabolic pathways to balance supply and demand and ultimately regulate cell and organ growth [53]. Furthermore, AMPK has been involved in the regulation of tumorigenesis [54]. The major kinase LKB1, upstream of AMPK, is a defective gene in Peutz-Jeghers syndrome. Considering that Peutz-Jeghers syndrome is a rare hereditary disease that is prone to tumor formation, suggests that the LKB1-AMPK axis will be an important cancer suppressor pathway [55–58]. Another study confirmed that AMPK signal activator metformin and AICAR can inhibit cancer cell growth and tumorigenesis [59]. Some studies suggested that reduced expression of AMPKα2 has been linked to primary breast cancer, gastric cancer and ovarian cancer but is rarely involved in NPC [60,61]. We observed that the expression of AMPKα1 is lower in NPC and negatively correlated with the expression of EBV-miR-BART1-5P. Moreover, from a metabolic standpoint, inactivation of AMPKα1 in both transformed and non-transformed cells facilitates conversion to aerobic glycolysis and increases glucose distribution to lipids [62]. On the other hand, AMPK activation inhibits downstream AKT, mTOR, HIF1a expression and inhibits glycolysis [63]. In this study, we found that EBV-miR-BART1-5P downregulated AMPKα1 expression, which could increase the expression of mTOR and HIF1a in NPC cells. These observations support that EBV-miR-BART1-5P mediated glycolysis and induced angiogenesis occurs by targeting AMPKα1 to activate the AMPK / mTOR / HIF1 pathway.

In summary, our results show that EBV-miR-BART1-5P has important roles in cancer cell glucose metabolism and angiogenesis by inhibiting AMPKα1, which provides a molecular basis for the regulation of AMPK/mTOR/HIF1 pathway. Our findings provide new insights into glycolysis and angiogenesis of NPC and new opportunities for the development of targeted NPC therapy in the future.

## Methods

### Ethics statement

The clinical processes for all the clinical tissues specimens were approved by the Ethics Committees of Zhongshan People’s Hospital. Informed written consent was obtained from all patients. And all patients were adult with independent morals or legal entitlements.

Animal experiments were approved by the Ethical Committee for Animal Research of the Southern Medical University (protocol number: 2011–020) and conducted based on the state guidelines from the Ministry of Science and Technology of China. White Leghorn chicken eggs with 9–10 days of embryonation were used for the chicken chorioallantoic membrane assay.

### Cell culture

2 EBV-negative NPC cell lines (HONE1 and HK1, previously provided by Professor S. W. Tsao, HKU), 6 EBV-negative epithelial cell lines (CNE1, 5-8F, 6-10B, SUNE1, HNE1 and CNE2) and HEK293T cells were obtained from the Cancer Research Institute, Southern Medical University, Guangzhou, China. The STR profiling of HONE1 cell line was showed in the supplementary file (STR profiling for HONE1). According to the International Cell Line Authentication Committee (ICLAC) database, CNE1, 5-8F, 6-10B, SUNE1, HNE1 and CNE2 cells may be contaminated by Hela cells. However, unlike Hela cells, which are resistant to EBV infection, CNE1 and CNE2 cells are susceptible to EBV infection in vitro[64]. So, in our study, we mainly focused on the effect of EBV-encoded miRNA-BART1 on host cell. This effect may be appropriate for all EBV-associated tumors Three EBV-positive NPC cell lines (C666-1, HONE1-EBV and HK1-EBV) and NP460, an immortalized human nasopharyngeal epithelial cell line, were kindly provided by Professor S. W. Tsao, University of Hong Kong. The STR profiling of these cells were conducted by Professor S. W. Tsao. [65]. NPC cell lines were cultured in PRMI-1640 (Invitrogen) supplemented with 10% fetal bovine serum (FBS) (Hyclone, Invitrogen), 100 U/ml penicillin and 100 ug/ml streptomycin. NP460, an immortalized human nasopharyngeal epithelial cell line, was cultured in defined KSFM medium supplemented with epidermal growth factor (Invitrogen, Carlsbad, SA). All cells were maintained in a humidified chamber with 5% CO_2_ at 37°C.

### Tissue specimens

55 primary NPC tissues (no treatment before biopsy) and 15 non-cancerous nasopharyngeal tissues were collected from patients at the Zhongshan People’s Hospital, Guangdong, China. All specimens were staged according to the TNM classification and used for qPCR and clinical analysis. Only those NPC samples that contained >80% of homogeneous cancer cells on frozen cross-sections visualized by haematoxylin-eosin staining were included in the study. The pathologic stage of all specimens was confirmed according to the 1992 Fuzhou NPC staging system of China.

### qRT-PCR

Total RNA was extracted from cells by Trizol reagent (Invitrogen), complementary DNA (cDNA) was synthesized with the PrimeScript RT reagent Kit (TaKaRa Bio, Inc., Shiga, Japan). PCR analyses were performed with SYBR PremixTag (TaKaRa). The primers used are shown in S2 Table. Small nuclear RNA RNU6B (U6 snRNA) and GAPDH expression were used for normalizing the expression of miRNA and mRNA, respectively. The qRT-PCR reactions for each sample were repeated three times in three independent experiments. The fold changes were calculated by using the relative quantification method (2^−ΔΔCt^).

### Lentivirus infection

Lentivirus (GV209, H1-MCS-CMV-EGFP) particles carrying EBV-miR-BART1-5P precursor (BART1-5P) or its flanking negative control sequence (NC) were constructed by GeneChem (Shanghai, China) and transduced into NPC cells and CNE1(a contaminated EBV negative epithelial cell line) following the manufacturer’s instructions. The virus-infected cells, being GFP positive, were sorted by a BD FACS Aria cell sorter 72h after transduction.

### Plasmid preparation and cell transfection

The expression vector GV230 containing the whole coding sequence of PTEN, AMPKα1 and the control vector GV170 were purchased from GeneChem (Shanghai, China). Both HONE-BART1-5P and CNE1-BART1-5P cells were transfected with 200ng plasmid DNA using Lipofectamine 2000 reagent (Invitrogen). 48 hours post transfection, the cells were harvested for qRT-PCR and western blotting analyses. The EBV-miR-BART1-5P mimic (5’-UCUUAGUGGAAGUGACGUGCUGUG-3’), EBV-miR-BART1-3P mimic (5’-UAGCACCGCUAUCCACUAUGUC-3’), EBV-miR-BART1-5P inhibitor(anti-miR, 2′-O-methyl modification) (5’-CACAGCACGUCACUUCCACUAAGA-3’), EBV-miR-BART1-3P inhibitor (anti-miR, 2′-O-methyl modification) (5’-GACAUAGUGGAUAGCGGUGCUA-3’) and associated nonspecific mimic (5’-UUGUACUACACAAAAGUACUG-3’) or inhibitor (5’-CAGUACUUUUGUGUAGUACAA-3’) controls were synthesized by GenePharma, Shanghai,China.

All cells were maintained in a humidified atmosphere of 95% air and 5% CO2 at 37°C, and seeded 24h prior to transfection. EBV-miR-BART1-5P, BART1-3P mimic, anti-miR, and their mock control were transfected into cells at a final concentration of 50 nmol/l using Lipofectamine 2000 (Invitrogen) in serum-free conditions. Six hours later, the medium was changed to fresh RPMI-1640 (Invitrogen) with 10% fetal bovine serum (Hyclone, Invitrogen).

### RNA deep-sequencing

Total RNA from CNE1-BART1 cells or mock control cells was extracted with Trizol Reagent (Invitrogen) according to the manufacturer’s introduction. RNA-deep sequencing was performed and analyzed in BGI-Shenzhen of China as previously described[31].

### Western blotting

Cell pellets were lysed in RIPA buffer containing protease (Sigma-Aldrich) and phosphatase inhibitors (Keygen, China), and the protein concentration was determined using the BCA assay (Beyotime, Beijing, China). Proteins were separated by a 10% SDS-PAGE gel, and blotted onto a polyvinylidene difluoride membrane (Milipore, Billerica, MA, USA). The membrane was probed with the first antibody listed in S1 Table and then with the peroxidase conjugated secondary antibody. GAPDH and β-actin were used as protein loading controls. Western blotting bands were visualized by the eECL Western Blot Kit (CWBIO Technology) and captured with a ChemiDoc CRSt Molecular Imager (Bio-Rad).

### Colony formation assay, EdU incorporation assay and cell cycle analyses

For the colony formation assay, NPC cells were seeded in duplicate in 6-well culture plates at a density of 100 cells/well. After incubation for 14 days at 37°C, colonies were washed twice with PBS and stained with hematoxylin solution. The colonies composed of more than 50 cells were counted under a microscope. All the experiments were repeated at least three times. For the EdU incorporation assay, proliferating NPC cells were examined using the Cell-Light EdU In Vitro Imaging Kit (RiboBio) according to the manufacturer’s protocol. FACS assays of NPC cells were performed after transfection with NC, anti-c, EBV-miR-BART1-5P mimics, inhibitor and/or PTEN plasmid, si-PTEN as previously described[66].

### In vivo tumorigenesis in nude mice

All nude mice (4–5 weeks old, female) were purchased from the Central Animal Facility of the Southern Medical University. To assess tumor growth, 100 ul of HONE1-BART1 cells or mock control cells (5x10^6^) were subcutaneously injected into the left or right side of the back of each mouse (six mice per group). The tumor sizes were measured regularly and calculated using the formula 0.52 x L x W^2^ where L and W are the long and short diameter of the tumor, respectively.

### In vivo matrigel plug assay

HONE1 cells transfected EBV-miR-BART1-5P (50nM) alone or co-transfected EBV-miR-BART1-5P and PTEN plasmid. Cells were exposed to serum-free media for 48 h. We then collected the supernatants and centrifuged them to remove cells. The conditioned media were then mixed with phenol-red-free Matrigel (2:3 proportion, total 0.5 ml; BD Biosciences). The mixture was then injected into each mouse subcutaneously (n = 3 per group). The mice were killed on day 8 and matrigel plug was examined for haemoglobin content using the QuantiChrom hemoglobin assay kit as per the manufacturer’s protocol (BioAssay Systems).

### In vitro angiogenesis assays

An in vitro endothelial tube formation was done as described previously[67]. Briefly, matrigel was added (50 uL) to each well of a 96-well plate and allowed to polymerize. HUVECs were suspended in medium at a density of 3×10^5^ cells/mL, and 0.1 mL of the cell suspension was added to each well coated with Matrigel. Cells were incubated for 12 hours at 37°C. The cells were then photographed, and branch points from 4 to 6 high-power fields (200x) were counted and averaged. The number of nodes (defined as when at least three cells formed a single point) per image was quantified.

### In vivo assessment of angiogenesis using chicken chorioallantoic membrane assay

For the chorioallantoic membrane (CAM) assay, white Leghorn chicken eggs (South China Agricultural University, Guangzhou, China) were incubated under routine conditions (constant humidity and 37°C) and a square window was opened in the egg shell at day 3 of incubation, to remove 3.5 mL of albumen and to detach the shell from the developing CAM. The window was sealed with a glass of the same size, and the eggs were returned to the incubator. Gelatin sponges were cut to a size of 1 mm^3^ and placed on the top of the CAM at day 8 under sterile conditions[68]. The sponges were then absorbed with 5 μL of low molecular weight heparin and cancer cells were implanted on the CAM surface to be tested. Sponges containing PBS were used as negative controls. CAMs were examined daily and photographed in ovo at day 12. The areas occupied by the vessel plexus were quantified using an IPP 5.0 image analysis program. The blood vessel density was expressed as the percentage of area occupied by the blood vessels of control over the whole area under the microscopic field[69].

### IHC staining

Paraffin sections prepared from in vivo experiments were applied to IHC staining for the detection of protein expression levels of mTOR, S6K1, VEGF, HIF-1α, HK2, GLUT1, LDHA and VEGF. The indirect streptavidin-peroxidase method was used. All antibodies used for IHC are listed in S1 Table. The stained results were reviewed and scored by two pathologists independently. The intensity of immunostaining was scored as negative (0), weak (1), medium (2) and strong (3). The extent of staining, defined as the percent of positive staining cells, was scored as 1 (≤10%), 2 (11–50%), 3 (51–75%) and 4 (>75%). An overall expression score, ranging from 0 to 12, was obtained by multiplying the score of intensity and that of extent. The final staining score was presented as negative (overall score of (0), 1+ (overall score of 1–3), 2+ (overall score of 4) or 3+ (overall score of ≥5).

### Dual luciferase assay

HEK293T cells (1 × 10^4^) were cultured in 24-well plates and co-transfected with 20 nM EBV-miR-BART1-5P mimic or NC, 5 ng of pRL-CMV Renilla luciferase reporter and 30 ng of luciferase reporter that contained the wild-type or mutant 3’UTR of AMPKα1. For antagonism experiments, cells were also co-transfected with 20 nM anti-miR or anti-C(anti-control). Transfections were performed in duplicate and repeated in three independent experiments. Forty-eight hours after transfection, the luciferase activities were analyzed with a Dual-Luciferase Reporter Assay System (Promega, Madison, WI, USA).

### Lactate production, glucose consumption and 2-NBDG uptake

Cells were cultured in DMEM without phenol red for 15 h, and the culture media was then harvested for measurement of lactate or glucose concentrations. Lactate levels were quantified using the Lactate Assay kit (BioVision, Mountain View, USA), glucose levels were determined by using a glucose assay kit (Sigma-Aldrich). All values were normalized to total protein levels (BCA Protein Assay Kit, Thermo Scientific, Waltham, USA). Recipient cells were labelled with 100uM 2-[N-(7-nitrobenz-2-oxa-1,3-diazol-4-yl) amino]-2-deoxy-D-glucose (2-NBDG) (Sigma-Aldrich) diluted in glucose-free media and incubated for 40 min at 37°C. 2-NBDG levels were determined for measurement of fluorescence intensity by a confocal microscope (Olympus FV1000, Tokyo, Japan).

### Statistical analysis

All experiments were performed in triplicate. Data shown are mean ± s.e.m. (unless otherwise specified) from at least three independent experiments. SPSS 19.0 software was used for statistical analyses. Differences were considered to be statistically significant at values of P<0.05 by Student’s t-test for two groups, one-way ANOVA (analysis of variance) analysis for multiple groups and parametric generalized linear model with random effects for tumor growth. Correlation was analyzed with two-tailed Spearman’s correlation analysis. Single, double and triple asterisks indicate a statistical significance of *P<0.05, **P<0.01 and ***P<0.001 respectively.

## Supporting information

S1 Fig (**A**) Lactate production, (**B**) glucose consumption in 7 EBV-negative epithelial cell lines (including NPC cells) after transfection EBV-miR-BART1-3P mimic or EBV-miR-BART1-5P mimic. The data were shown as the mean ± s.e.m. (*P<0.05, **P<0.01 and ***P<0.001).
(AI) Click here for additional data file.

S2 Fig EBV-negative NPC cell lines (GFP+) stably expressing EBV-miR-BART1-5P are generated using lentivirus infection. Two stable cell lines were indicated as HONE1-BART1-5P (**A left**) and CNE1-BART1-5P (**B left**) respectively. qPCR showed EBV-miR-BART1-5P expression levels in HONE1-BART1-5P (**A right**), CNE1-BART1-5P (**B right**) cells and their corresponding control cells (HONE1-NC and CNE1-NC) compared with NPC tissues (5 samples were pooled). Data were shown as the mean ± SEM. ***P < 0.001.
(AI) Click here for additional data file.

S3 Fig (**A**) Cellular ATP level in Hk1 and HONE1 cells after transfection EBV-miR-BART1-5P mimic alone or co-transfection EBV-miR-BART1-5P mimic and PTEN plasmid. (**B**) Cellular ATP level in Hk1-BART1-5P and HONE1-BART1-5P cells after transfection anti-miR alone or co-transfection anti-miR and si-PTEN. Anti-Control abbreviated anti-c. Anti-EBV-miR-BART1-5P abbreviated anti-miR. The data were shown as the mean ± s.e.m. (*P<0.05, **P<0.01 and ***P<0.001). The cellular levels of glucose-6-phosphate and ATP were measured using a Glucose-6-phosphate Fluorometric Assay kit (Cayman, Michigan, USA) and a CellTiter-Glo Luminescent Cell Viability Assay (Promega), respectively. All values were normalized to total protein levels.
(PPTX)

S4 Fig CAM angiogenesis was performed with NPC cells overexpressing (**A**) or inhibiting (**B**) EBV-miR-BART1-5P. Representative images of new blood vessel formation are shown(left), new blood vessels were counted under a dissecting microscope(right). VA = Vascular area CAM = Chorioallantoic membrane area (mm^2^).
(PPTX)

S5 Fig FACS assays of NPC cells, HK1 cells (**A and B left**) and HONE1 cells (**A and B right**) were performed after transfection with NC, anti-c, EBV-miR-BART1-5P mimics, inhibitor and/or PTEN plasmid, si-PTEN as indicated. Anti-Control abbreviated anti-c. Anti-EBV-miR-BART1-5P abbreviated anti-miR. The data were shown as the mean ± s.e.m (*P<0.05, **P<0.01 and ***P<0.001).
(PPTX)

S6 Fig The levels of AMPKα1 was evaluated by immunohistochemistry assay in NPC and NP tissue specimens. Magnification, ×400. (20 primary NPC tissues and 10 non-cancerous nasopharyngeal tissues were collected from patients at the Zhongshan People’s Hospital, Guangdong, China. The clinical processes were approved by the Ethics Committees of Zhongshan People’s Hospital.
(PPTX)

S7 Fig (**A**) Tumorigenicity of HONE1-EBV-B1-antagomiR cells was markedly reduced in vivo, n = 6/group. (**B**) Tumour volume was periodically measured for each mouse and tumour growth curves was plotted. Parametric generalized linear model with random effects.
(PPTX)

S8 Fig CAM angiogenesis detected in HK1-BART1-5P cells after transfection AMPKα1 plasmid, PTEN plasmid, AICAR and Dorsomorphin, respectively. The data were shown as the mean ± s.e.m. (*P<0.05, **P<0.01 and ***P<0.001). AMPK agonist: AICAR, AMPK inhibitor: Dorsomorphin.
(PPTX)

S9 Fig VEGF, HIF-1α and GLUT1 protein expression levels in Hk1-BART1-5P (**A**) and HONE1-BART1-5P (**B**) cells treated with AMPKα1 plasmid, PTEN plasmid AICAR and Dorsomorphin, respectively. β-actin was used as a loading control.
(PPTX)

S10 Fig mTOR, VEGF, HIF-1α and GLUT1 protein expression levels in C666-1 cells treated with anti-control, anti-miR, si-AMPKα1, si-PTEN, AICAR and Dorsomorphin, respectively. β-actin was used as a loading control. AMPK agonist: AICAR, AMPK inhibitor: Dorsomorphin.
(PPTX)

S11 Fig CAM angiogenesis detected in C666-1 cells treated with anti-control, anti-miR, si-AMPKα1, si-PTEN, AICAR and Dorsomorphin, respectively. The data were shown as the mean ± s.e.m. (*P<0.05, **P<0.01 and ***P<0.001).
(PPTX)

S12 Fig (**A**) AMPKα1, mTOR, p- mTOR, VEGF, HIF-1α, GLUT1 and LDHA protein expression levels in C666-1 and HK1 cells. (**B**) AMPKα1, mTOR, p- mTOR, VEGF, HIF-1α, GLUT1 and LDHA protein expression levels in NP460 cells after transfection NC or EBV-miR-BART1-5P. β-actin was used as a loading control.
(PPTX)

S13 Fig (**A**) EBV-miR-BART1-5P in three EBV-positive NPC cell lines (C666-1, HONE1-EBV, and HK1-EBV) and compared it with NPC clinical samples by qRT-PCR. (**B**) Down-regulation of the expression of EBV-miR-BART1-5P in HONE1-EBV cells after transfection of BART1-5P inhibitory oligonucleotide by qRT-PCR. he data were shown as the mean ± s.e.m. (*P<0.05, **P<0.01 and ***P<0.001).
(PPTX)

S1 Table The information of antibodies used in the present study. (DOCX)

S2 Table Primer sequences used in the present study. (DOCX)

S3 Table The information of clinical samples for clinical data analysis. (DOCX)

S4 Table Protein quantification by western blot. (XLSX)

S1 Data The STR profiling data for HONE1 cell line. (PDF)
