# Correction: EBV-miR-BART1-5P activates AMPK/mTOR/HIF1 pathway via a PTEN independent manner to promote glycolysis and angiogenesis in nasopharyngeal carcinoma

**DOI:** 10.1371/journal.ppat.1008532

**Published:** 2020-04-27

**Authors:** Xiaoming Lyu, Jianguo Wang, Xia Guo, Gongfa Wu, Yang Jiao, Oluwasijibomi Damola Faleti, Pengfei Liu, Tielian Liu, Yufei Long, Tuotuo Chong, Xu Yang, Jing Huang, Mingliang He, Chi Man Tsang, Sai Wah Tsao, Qian Wang, Qiang Jiang, Xin Li

The following information is missing from the Funding statement: This work was also supported by the Shenzhen Key Laboratory of Viral Oncology (ZDSYS201707311140430).
