# Supplementary figures and images for: EBV-miR-BART1-5P activates AMPK/mTOR/HIF1 pathway via a PTEN independent manner to promote glycolysis and angiogenesis in nasopharyngeal carcinoma
Source: PLoS Pathog. 2018 Dec 17;14(12):e1007484. doi: 10.1371/journal.ppat.1007484 (PMC6312352; doi:10.1371/journal.ppat.1007484)

## Slide 1
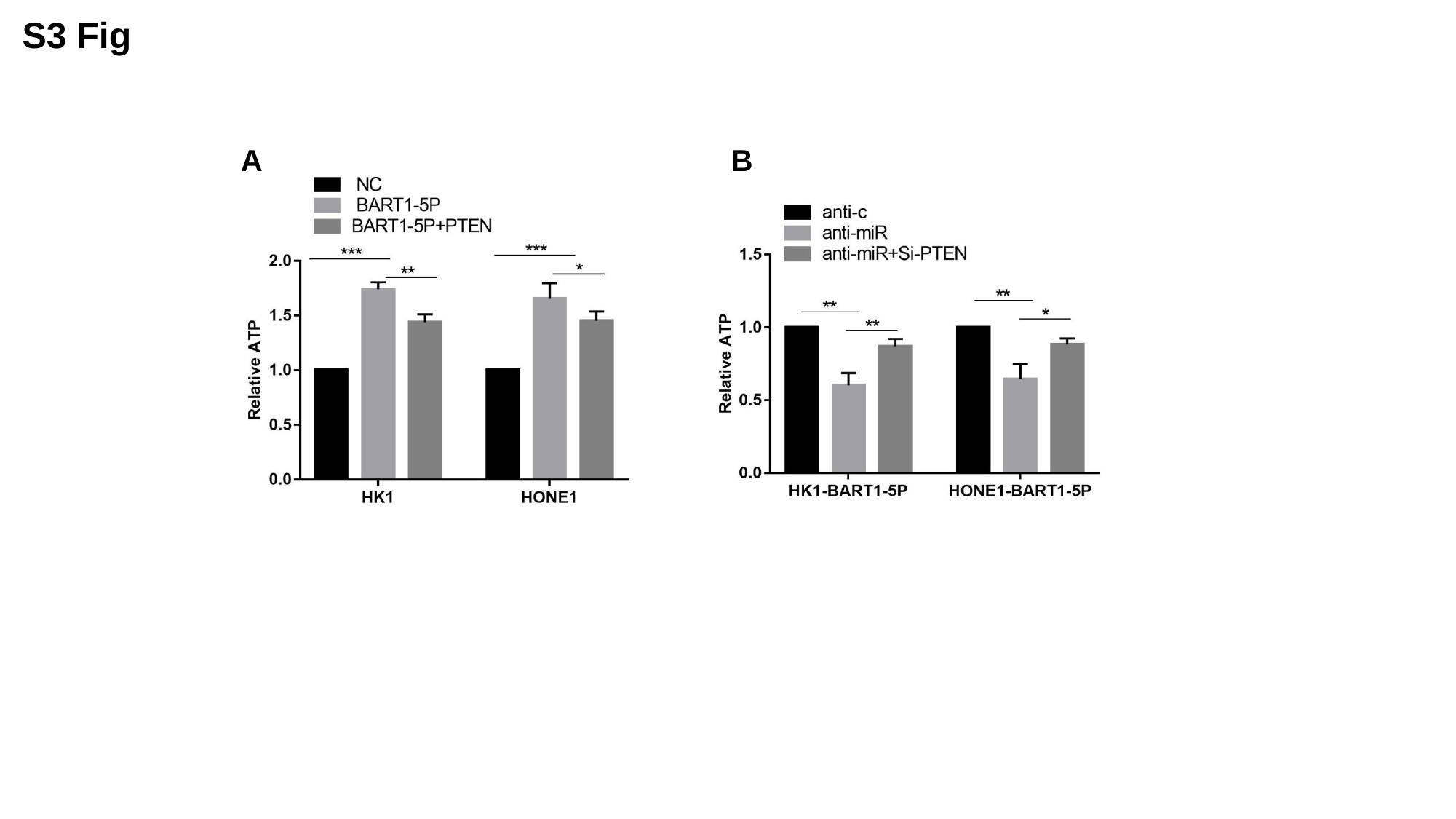

S3 Fig
A
B

Supplement: S3 Fig — (A) Cellular ATP level in Hk1 and HONE1 cells after transfection EBV-miR-BART1-5P mimic alone or co-transfection EBV-miR-BART1-5P mimic and PTEN plasmid. (B) Cellular ATP level in Hk1-BART1-5P and HONE1-BART1-5P cells after transfection anti-miR alone or co-transfection anti-miR and si-PTEN. Anti-Control abbreviated anti-c. Anti-EBV-miR-BART1-5P abbreviated anti-miR. The data were shown as the mean ± s.e.m. (*P<0.05, **P<0.01 and ***P<0.001). The cellular levels of glucose-6-phosphate and ATP were measured using a Glucose-6-phosphate Fluorometric Assay kit (Cayman, Michigan, USA) and a CellTiter-Glo Luminescent Cell Viability Assay (Promega), respectively. All values were normalized to total protein levels. (PPTX) [file ppat.1007484.s003.pptx]

## Slide 1
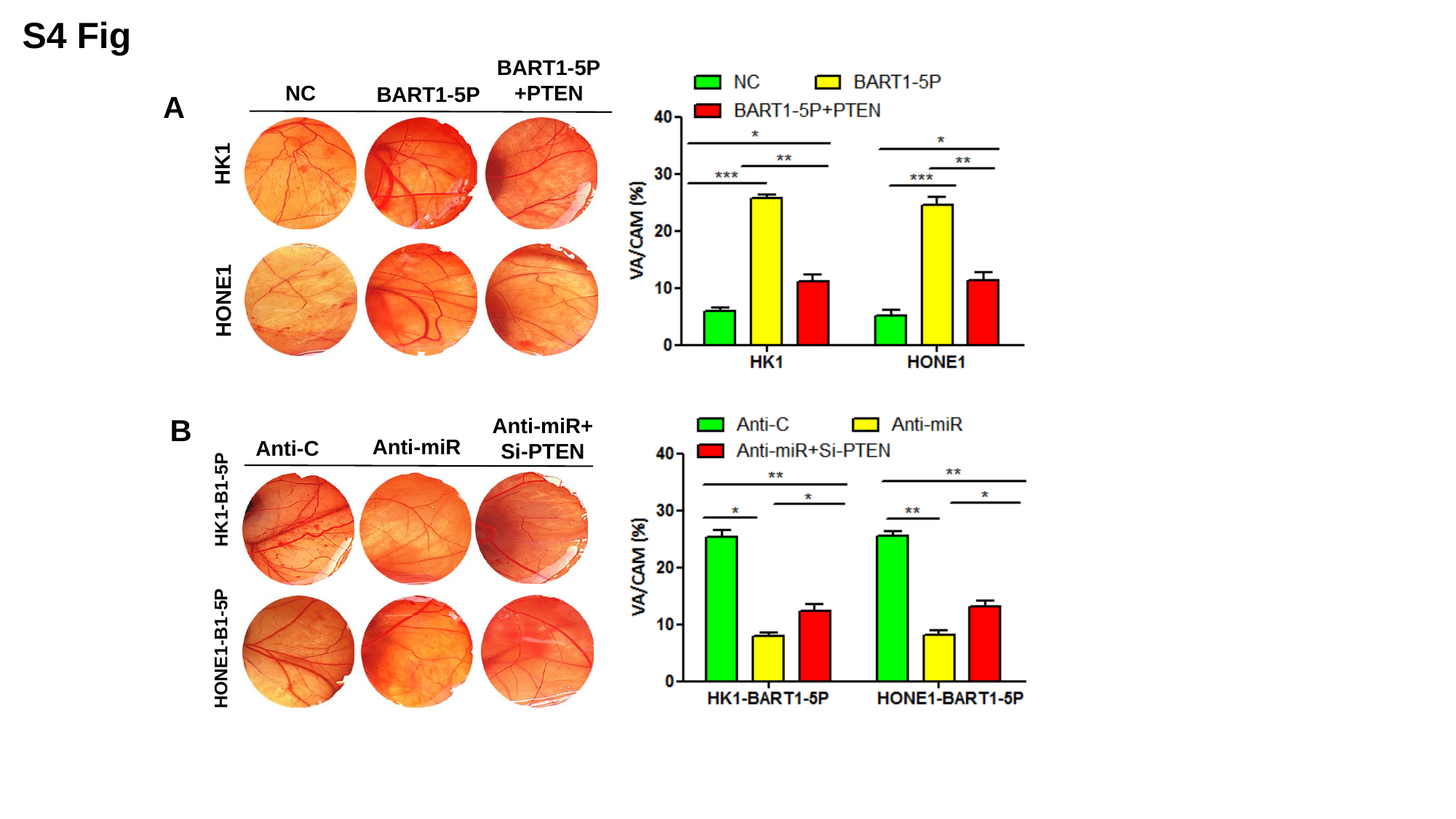

S4 Fig
BART1-5P
+PTEN
NC
BART1-5P
HK1
HONE1
A
Anti-miR+
Si-PTEN
Anti-miR
Anti-C
HK1-B1-5P
HONE1-B1-5P
B

Supplement: S4 Fig — CAM angiogenesis was performed with NPC cells overexpressing (A) or inhibiting (B) EBV-miR-BART1-5P. Representative images of new blood vessel formation are shown(left), new blood vessels were counted under a dissecting microscope(right). VA = Vascular area CAM = Chorioallantoic membrane area (mm2). (PPTX) [file ppat.1007484.s004.pptx]

## Slide 1
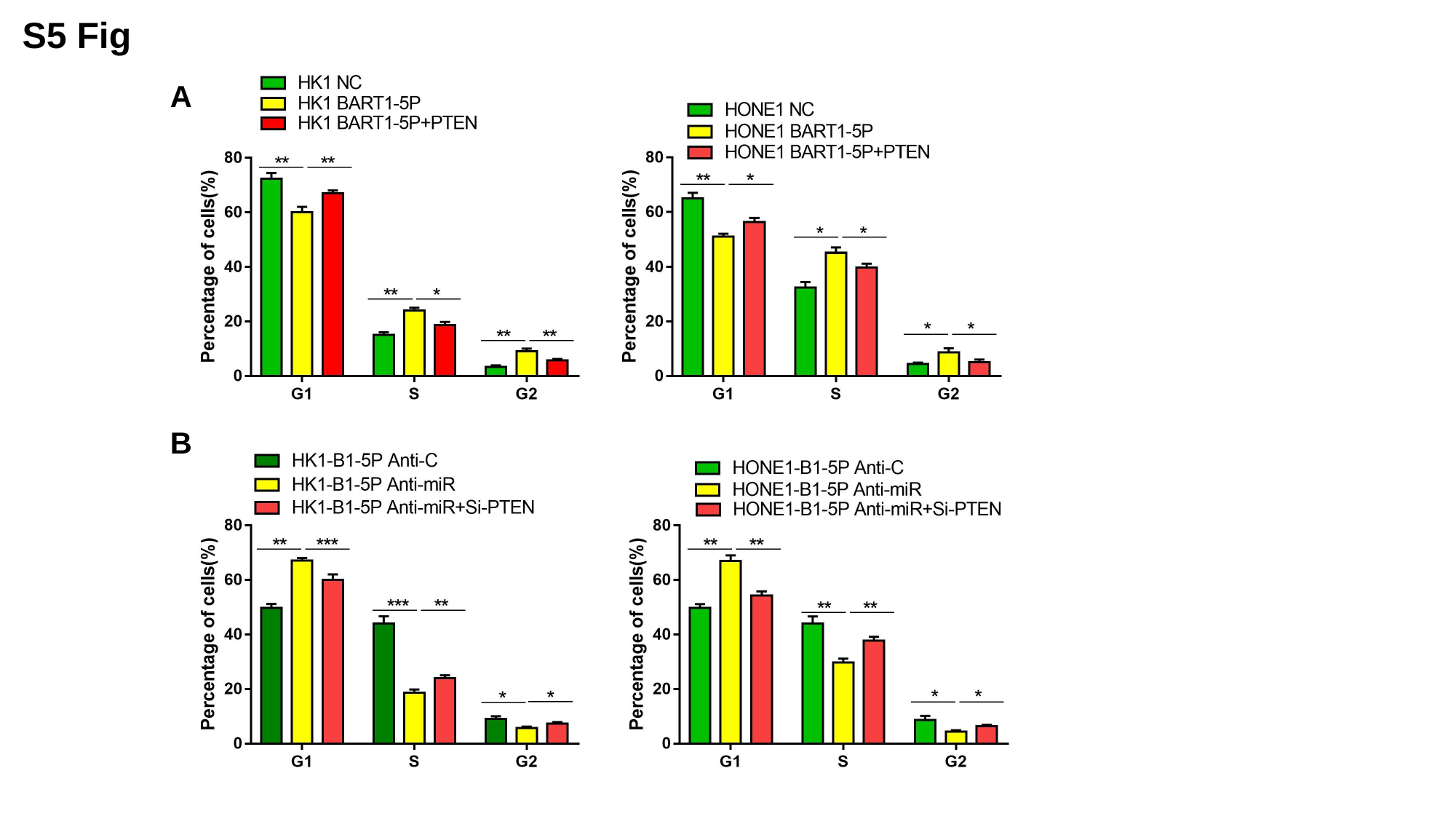

S5 Fig
A
B

Supplement: S5 Fig — FACS assays of NPC cells, HK1 cells (A and B left) and HONE1 cells (A and B right) were performed after transfection with NC, anti-c, EBV-miR-BART1-5P mimics, inhibitor and/or PTEN plasmid, si-PTEN as indicated. Anti-Control abbreviated anti-c. Anti-EBV-miR-BART1-5P abbreviated anti-miR. The data were shown as the mean ± s.e.m (*P<0.05, **P<0.01 and ***P<0.001). (PPTX) [file ppat.1007484.s005.pptx]

## Slide 1
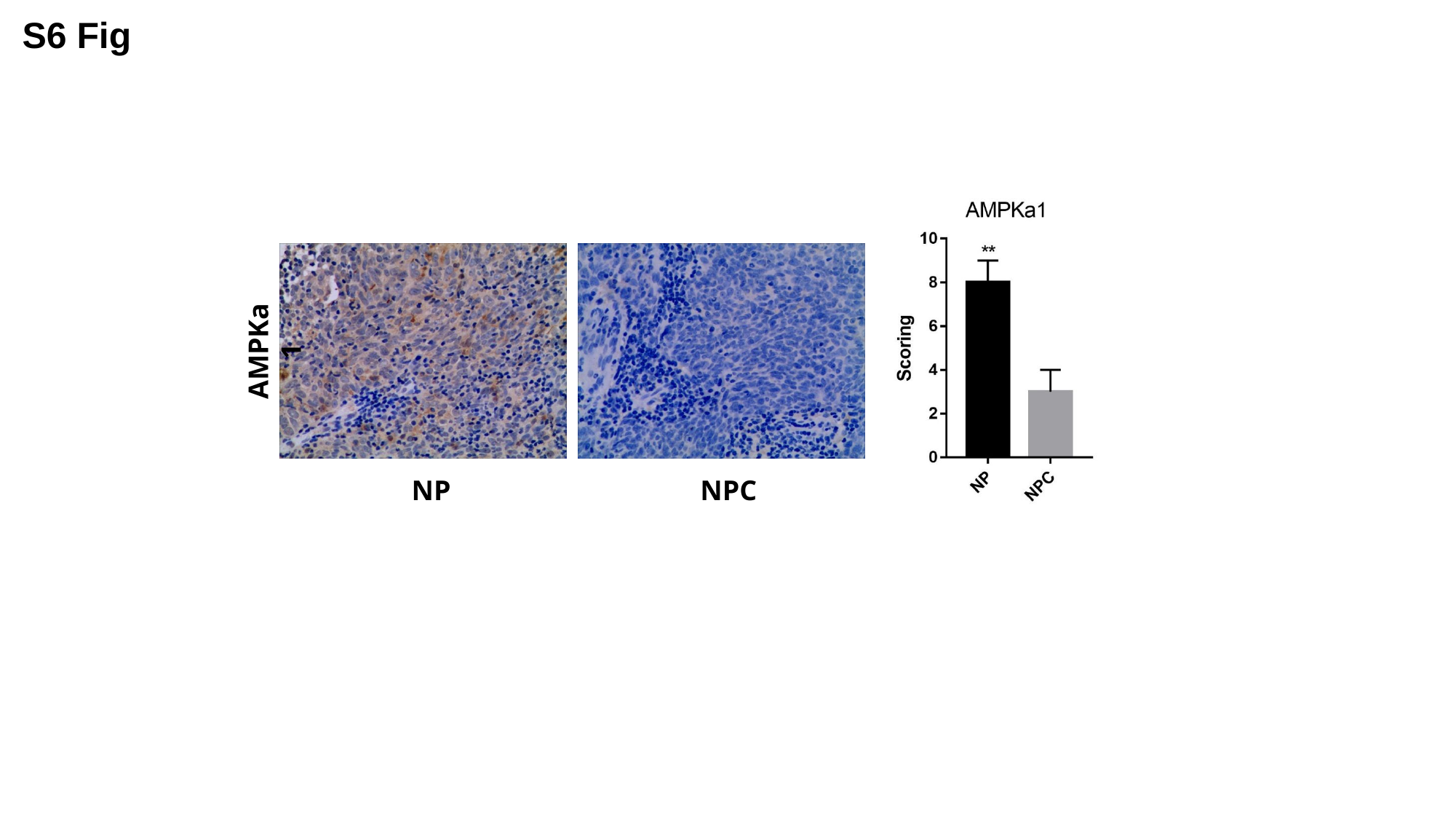

S6 Fig
AMPKa1
NP
NPC

Supplement: S6 Fig — Magnification, ×400. (20 primary NPC tissues and 10 non-cancerous nasopharyngeal tissues were collected from patients at the Zhongshan People’s Hospital, Guangdong, China. The clinical processes were approved by the Ethics Committees of Zhongshan People’s Hospital. (PPTX) [file ppat.1007484.s006.pptx]

## Slide 1
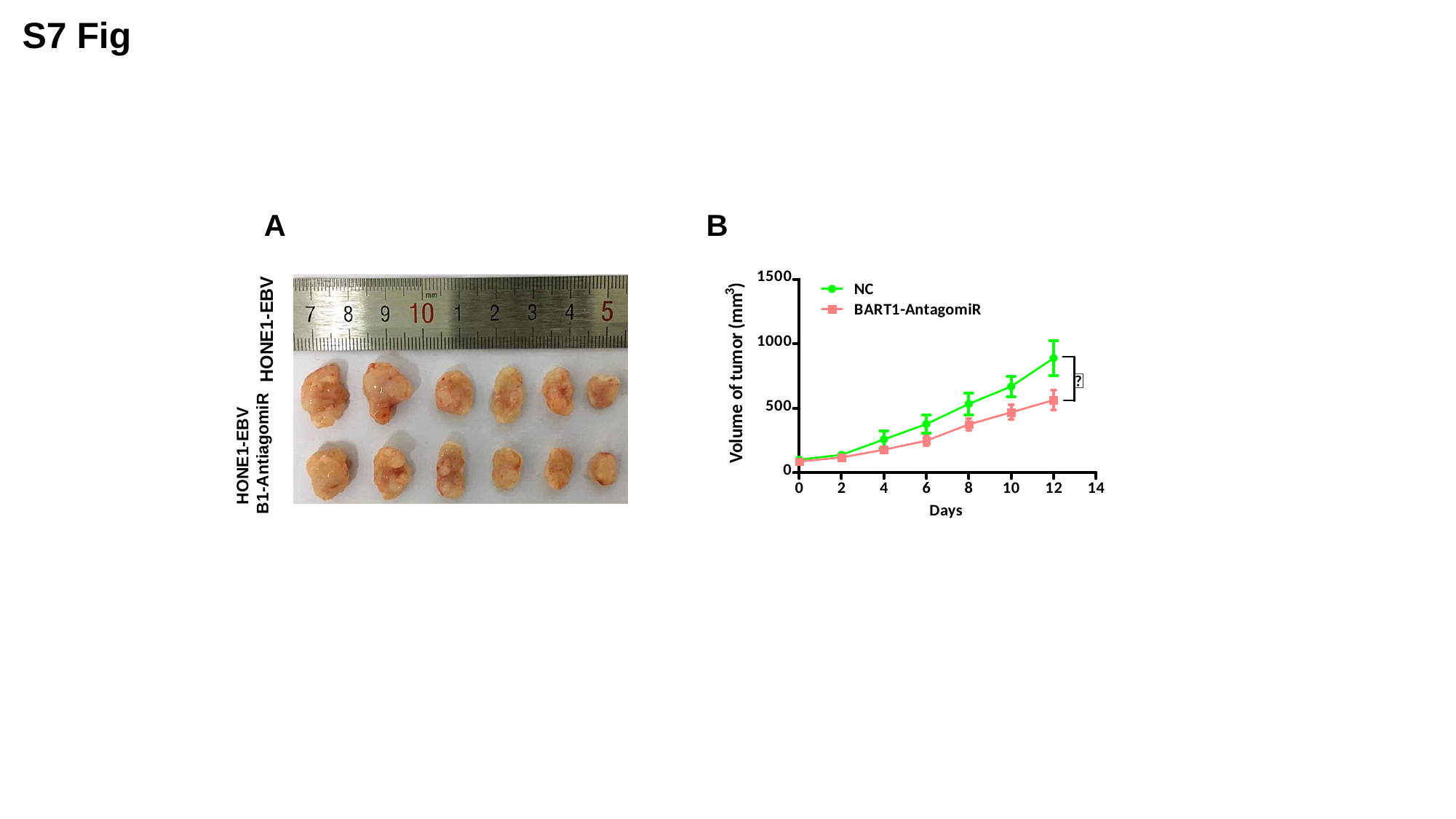

S7 Fig
A
B
HONE1-EBV
HONE1-EBV
B1-AntiagomiR

Supplement: S7 Fig — (A) Tumorigenicity of HONE1-EBV-B1-antagomiR cells was markedly reduced in vivo, n = 6/group. (B) Tumour volume was periodically measured for each mouse and tumour growth curves was plotted. Parametric generalized linear model with random effects. (PPTX) [file ppat.1007484.s007.pptx]

## Slide 1
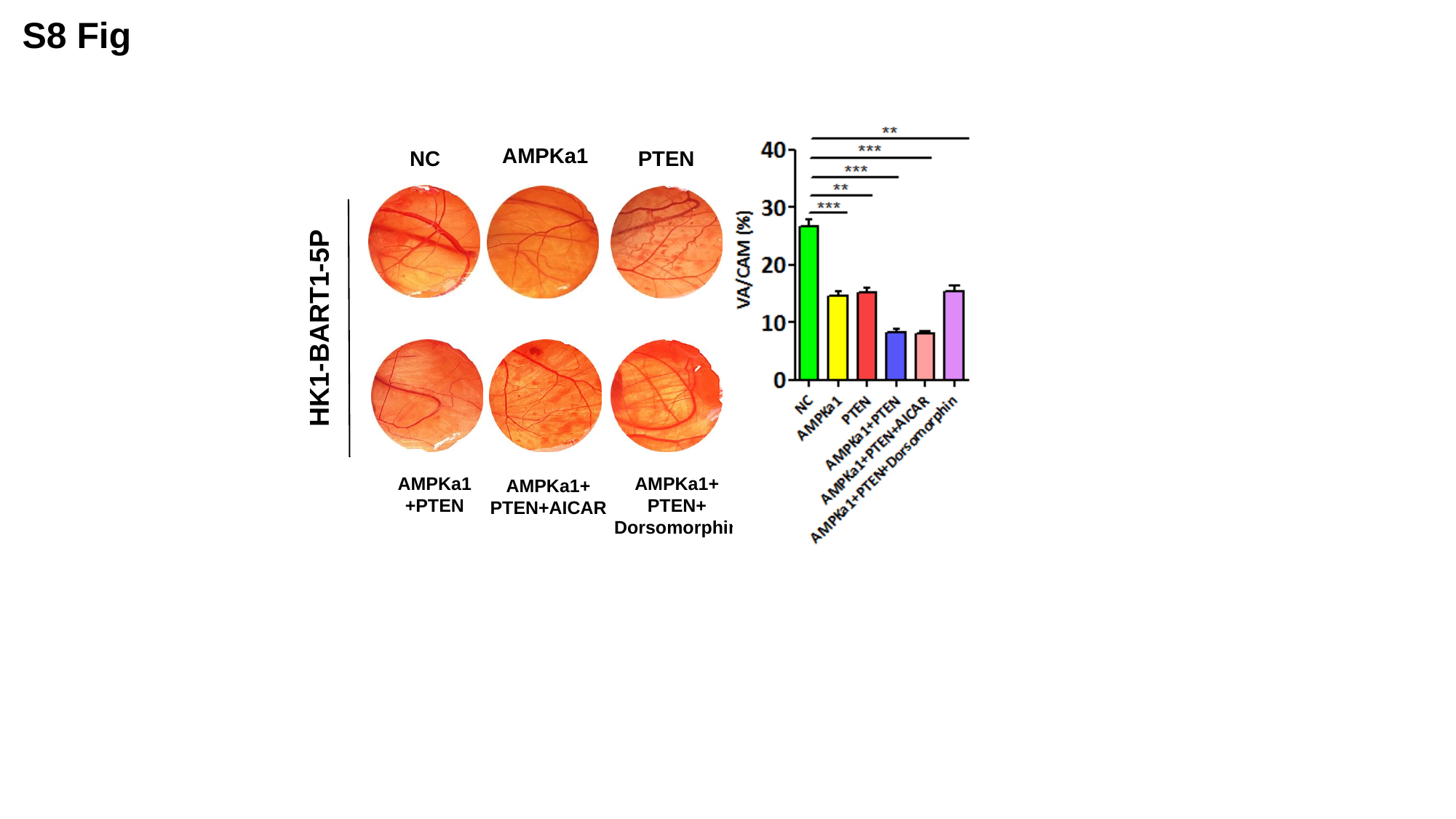

S8 Fig
AMPKa1
PTEN
NC
HK1-BART1-5P
AMPKa1
+PTEN
AMPKa1+
PTEN+
Dorsomorphin
AMPKa1+
PTEN+AICAR

Supplement: S8 Fig — The data were shown as the mean ± s.e.m. (*P<0.05, **P<0.01 and ***P<0.001). AMPK agonist: AICAR, AMPK inhibitor: Dorsomorphin. (PPTX) [file ppat.1007484.s008.pptx]

## Slide 1
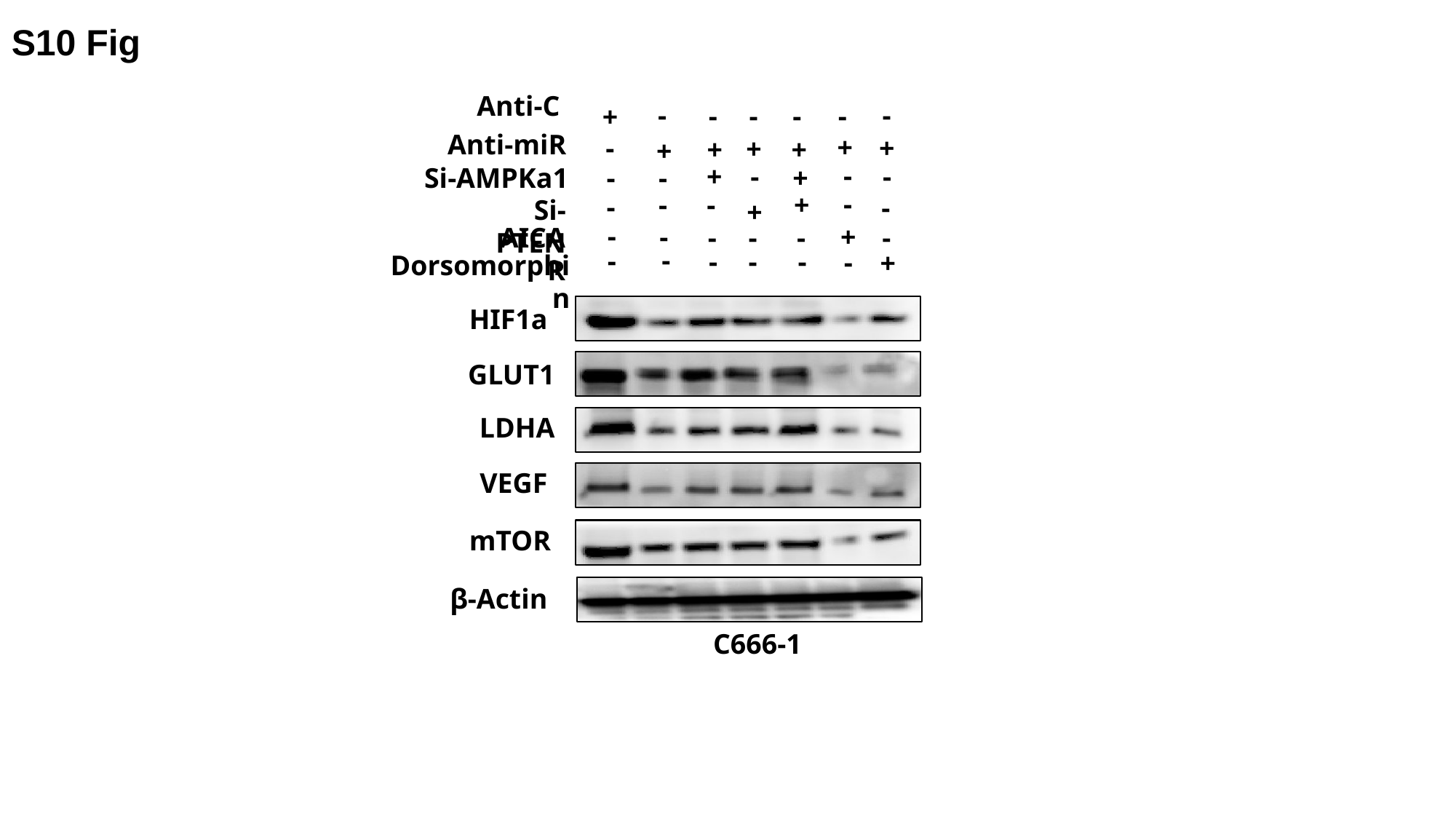

S10 Fig
Anti-C
-
-
-
-
-
-
+
Anti-miR
+
+
-
+
+
+
+
-
-
+
-
Si-AMPKa1
-
+
-
-
-
-
+
-
-
Si-PTEN
+
-
+
-
-
-
-
AICAR
-
-
-
-
-
-
-
+
Dorsomorphin
HIF1a
GLUT1
LDHA
VEGF
mTOR
β-Actin
C666-1

Supplement: S10 Fig — β-actin was used as a loading control. AMPK agonist: AICAR, AMPK inhibitor: Dorsomorphin. (PPTX) [file ppat.1007484.s010.pptx]

## Slide 1
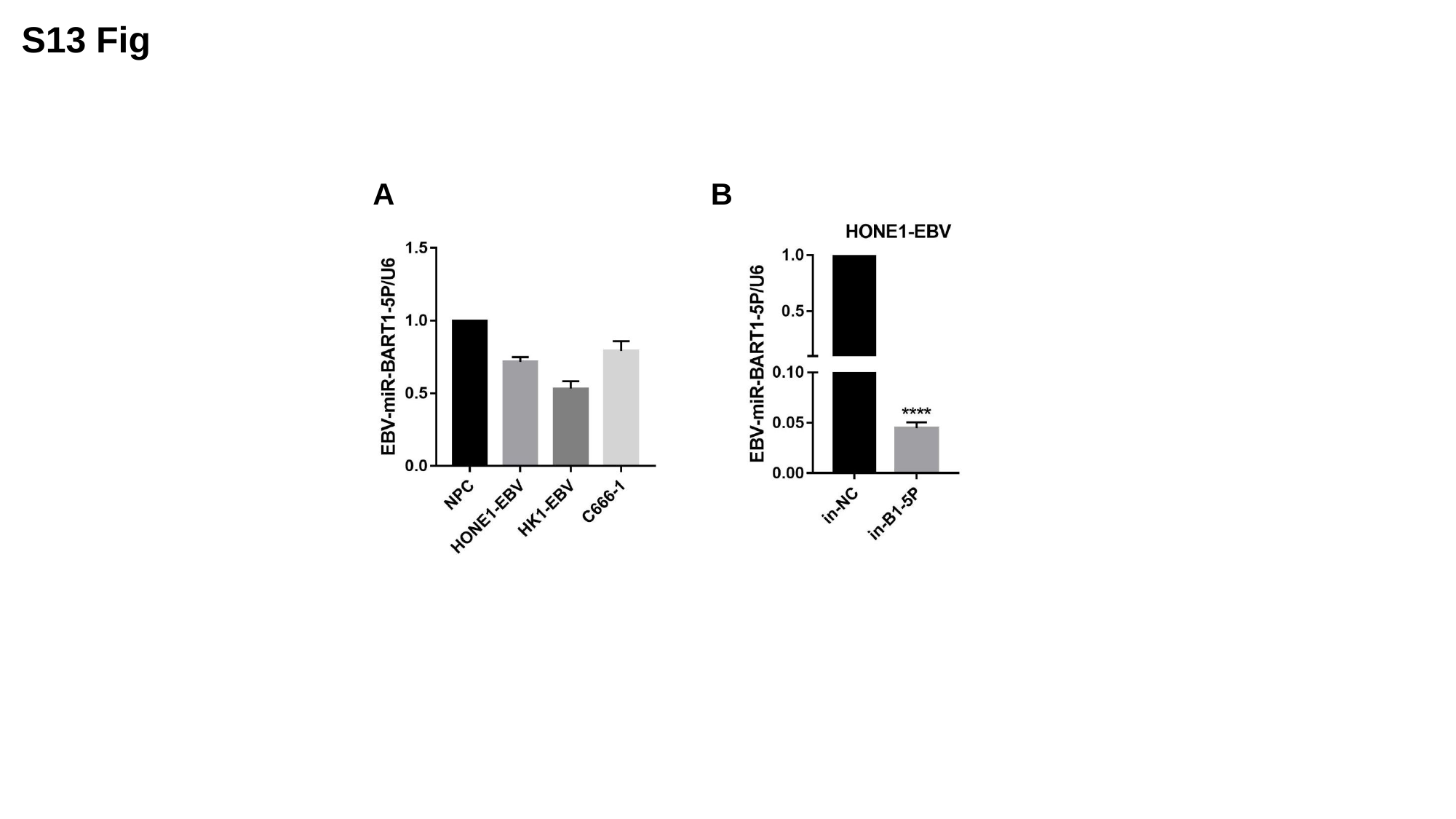

S13 Fig
A
B

Supplement: S13 Fig — (A) EBV-miR-BART1-5P in three EBV-positive NPC cell lines (C666-1, HONE1-EBV, and HK1-EBV) and compared it with NPC clinical samples by qRT-PCR. (B) Down-regulation of the expression of EBV-miR-BART1-5P in HONE1-EBV cells after transfection of BART1-5P inhibitory oligonucleotide by qRT-PCR. he data were shown as the mean ± s.e.m. (*P<0.05, **P<0.01 and ***P<0.001). (PPTX) [file ppat.1007484.s013.pptx]
