# Supplementary material for: EBV-miR-BART1-5P activates AMPK/mTOR/HIF1 pathway via a PTEN independent manner to promote glycolysis and angiogenesis in nasopharyngeal carcinoma
Source: PLoS Pathog. 2018 Dec 17;14(12):e1007484. doi: 10.1371/journal.ppat.1007484 (PMC6312352; doi:10.1371/journal.ppat.1007484)
Supplement: S9 Fig — VEGF, HIF-1α and GLUT1 protein expression levels in Hk1-BART1-5P (A) and HONE1-BART1-5P (B) cells treated with AMPKα1 plasmid, PTEN plasmid AICAR and Dorsomorphin, respectively. β-actin was used as a loading control. (PPTX) [file ppat.1007484.s009.pptx]

## Slide 1
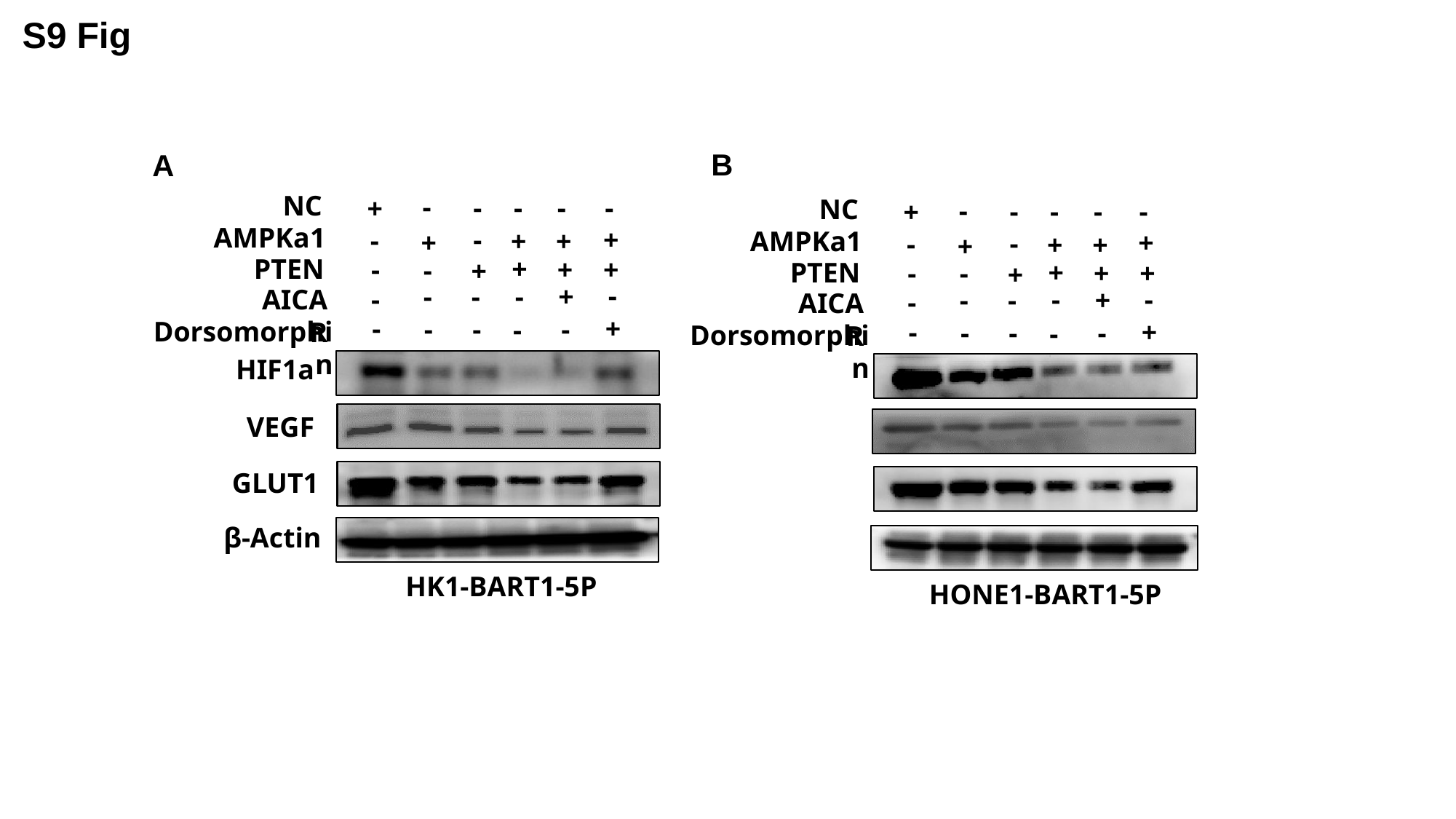

S9 Fig
B
A
NC
-
-
-
-
-
+
AMPKa1
+
-
-
+
+
+
PTEN
+
-
+
+
-
+
-
-
-
-
+
-
AICAR
-
+
-
-
-
-
Dorsomorphin
HIF1a
VEGF
GLUT1
β-Actin
HK1-BART1-5P
NC
-
-
-
-
-
+
AMPKa1
+
-
-
+
+
+
PTEN
+
-
+
+
-
+
-
-
-
-
+
-
AICAR
-
+
-
-
-
-
Dorsomorphin
HONE1-BART1-5P
