# Supplementary material for: EBV-miR-BART1-5P activates AMPK/mTOR/HIF1 pathway via a PTEN independent manner to promote glycolysis and angiogenesis in nasopharyngeal carcinoma
Source: PLoS Pathog. 2018 Dec 17;14(12):e1007484. doi: 10.1371/journal.ppat.1007484 (PMC6312352; doi:10.1371/journal.ppat.1007484)
Supplement: S11 Fig — The data were shown as the mean ± s.e.m. (*P<0.05, **P<0.01 and ***P<0.001). (PPTX) [file ppat.1007484.s011.pptx]

## Slide 1
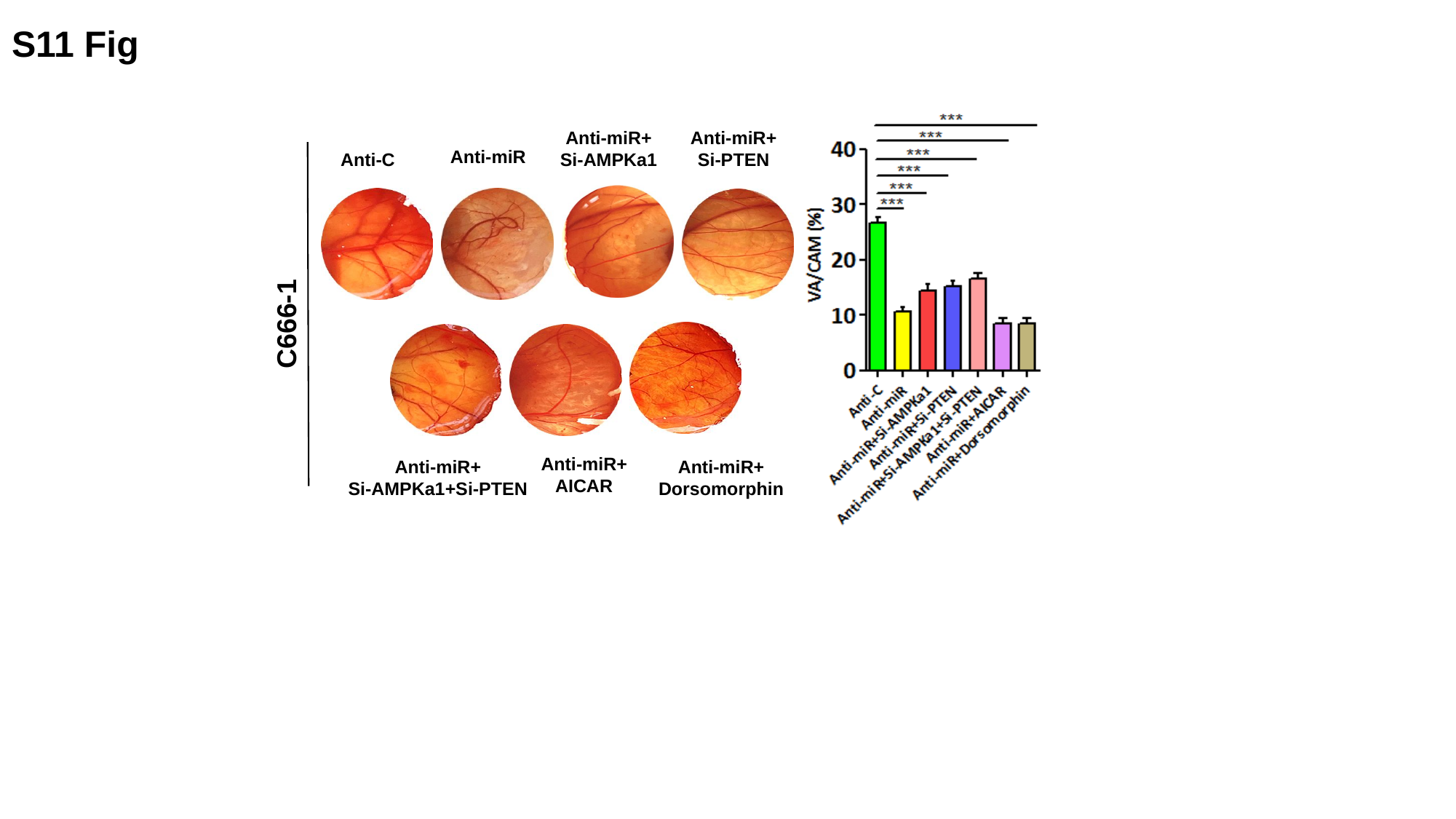

S11 Fig
Anti-miR+
Si-AMPKa1
Anti-miR+
Si-PTEN
Anti-miR
Anti-C
C666-1
Anti-miR+
AICAR
Anti-miR+
Si-AMPKa1+Si-PTEN
Anti-miR+
Dorsomorphin
