# Supplementary material for: EBV-miR-BART1-5P activates AMPK/mTOR/HIF1 pathway via a PTEN independent manner to promote glycolysis and angiogenesis in nasopharyngeal carcinoma
Source: PLoS Pathog. 2018 Dec 17;14(12):e1007484. doi: 10.1371/journal.ppat.1007484 (PMC6312352; doi:10.1371/journal.ppat.1007484)
Supplement: S12 Fig — (A) AMPKα1, mTOR, p- mTOR, VEGF, HIF-1α, GLUT1 and LDHA protein expression levels in C666-1 and HK1 cells. (B) AMPKα1, mTOR, p- mTOR, VEGF, HIF-1α, GLUT1 and LDHA protein expression levels in NP460 cells after transfection NC or EBV-miR-BART1-5P. β-actin was used as a loading control. (PPTX) [file ppat.1007484.s012.pptx]

## Slide 1
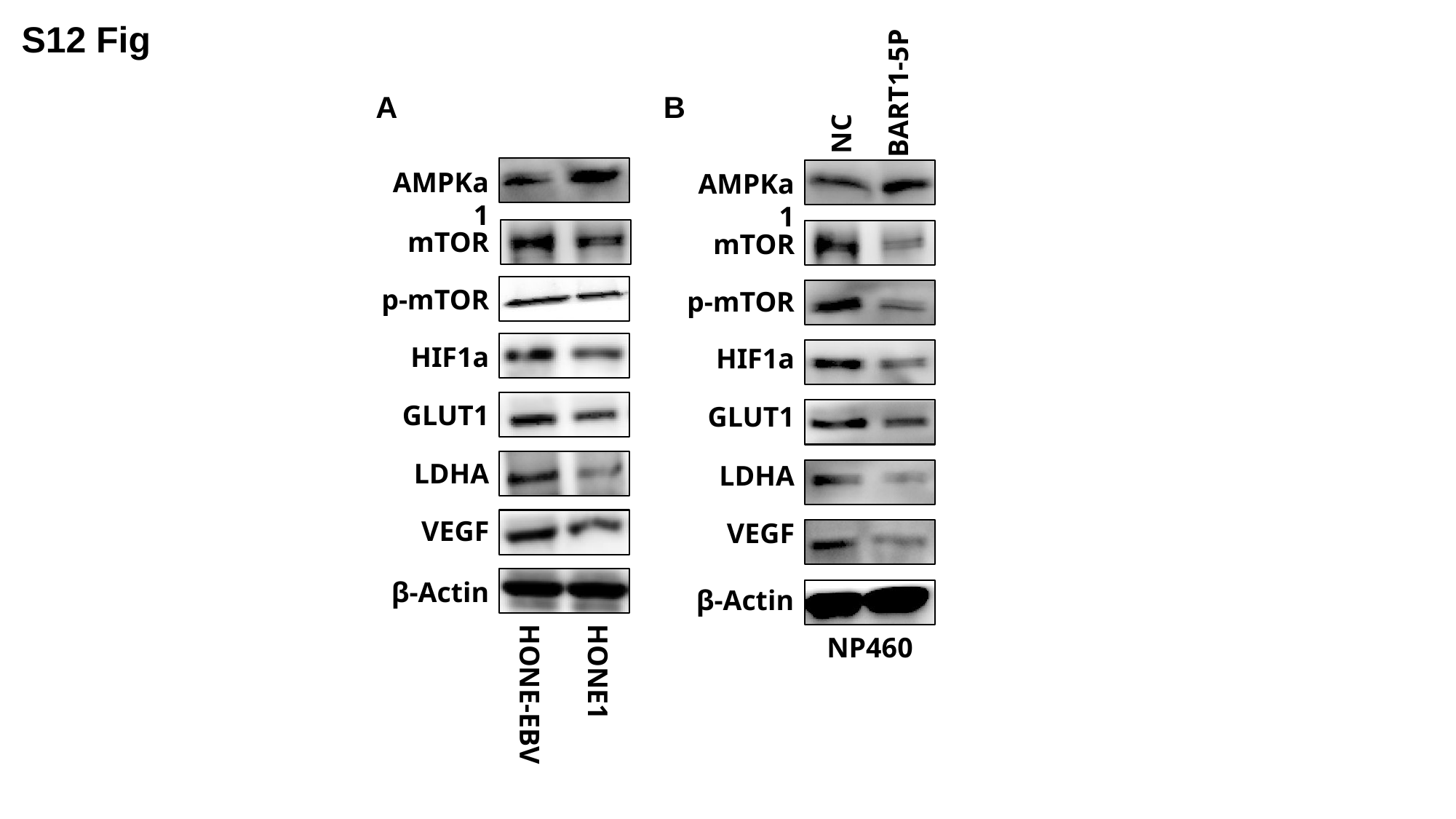

S12 Fig
BART1-5P
NC
AMPKa1
mTOR
p-mTOR
HIF1a
GLUT1
LDHA
VEGF
β-Actin
NP460
A
B
AMPKa1
mTOR
p-mTOR
HIF1a
GLUT1
LDHA
VEGF
β-Actin
HONE1
HONE-EBV
