# Supplementary material for: EBV-miR-BART1-5P activates AMPK/mTOR/HIF1 pathway via a PTEN independent manner to promote glycolysis and angiogenesis in nasopharyngeal carcinoma
Source: PLoS Pathog. 2018 Dec 17;14(12):e1007484. doi: 10.1371/journal.ppat.1007484 (PMC6312352; doi:10.1371/journal.ppat.1007484)
Supplement: S1 Table — (DOCX) [file ppat.1007484.s014.docx]

| **S1 Table** The information of antibodies used in the present study | | | | |
| --- | --- | --- | --- | --- |
| Antibodies | Cat. No | Company | Molecular weight | Dilution (WB/IHC) |
| PTEN(D375) pAb | BS1305 | Bioworld | 54KDa | 1:800(WB) |
| HIF1a | 20960-1-AP | Proteintech | 120kDa | 1:600(WB)/1:100(IHC) |
| HK2 | 22029-1-AP | Proteintech | 102kDa | 1:3000(WB)/1:50(IHC) |
| LDHA | 19987-1-AP | Proteintech | 37kDa | 1:5000(WB)/1:100(IHC) |
| GLUT1 | 66290-1-Ig | Proteintech | 45-55KDa | 1:1000(WB)/1:100(IHC) |
| P27 | 25614-1-AP | PTG | 27KDa | 1:500(WB) |
| P21 | 2947 | CST | 21KDa | 1:1000(WB) |
| CCND1 | Ab134175 | abcam | 34KDa | 1:1000(WB) |
| Ki-67 | Ab16667 | abcam | - | 1:100(IHC) |
| mTOR | 04-385 | Millipore | 289KDa | 1:1000(WB)/1:250(IHC) |
| p-mTOR | 5536 | CST | 289KDa | 1:1000(WB) |
| AMPKα1 | Ab32047 | abcam | 63KDa | 1:3000(WB)/1:250(IHC) |
| CD31 | Ab38346 | abcam | - | 1:50(IHC) |
| S6K1 | Ab32539 | abcam | 70 KDa | 1:8000(WB) |
| β-actin | Sc-1616 | Santa | 43KDa | 1:1000(WB) |
| GAPDH | P30008 | abmart | 37KDa | 1:1000(WB) |
