# Supplementary material for: EBV-miR-BART1-5P activates AMPK/mTOR/HIF1 pathway via a PTEN independent manner to promote glycolysis and angiogenesis in nasopharyngeal carcinoma
Source: PLoS Pathog. 2018 Dec 17;14(12):e1007484. doi: 10.1371/journal.ppat.1007484 (PMC6312352; doi:10.1371/journal.ppat.1007484)
Supplement: S2 Table — (DOCX) [file ppat.1007484.s015.docx]

| **S2 Table** Primer sequences used in the present study | | |
| --- | --- | --- |
| Gene | Primer | Sequence |
| AMPKα1 | Forward | 5'- TTGAAACCTGAAAATGTCCTGCT-3' |
|  | Reverse | 5'- GGTGAGCCACAACTTGTTCTT-3' |
| GAPDH | Forward | 5'-GCCACATCGCTCAGACACCA-3' |
|  | Reverse | 5'-CTCAGCCTTGACGGTGCCAT-3' |
