# Supplementary material for: EBV-miR-BART1-5P activates AMPK/mTOR/HIF1 pathway via a PTEN independent manner to promote glycolysis and angiogenesis in nasopharyngeal carcinoma
Source: PLoS Pathog. 2018 Dec 17;14(12):e1007484. doi: 10.1371/journal.ppat.1007484 (PMC6312352; doi:10.1371/journal.ppat.1007484)
Supplement: S3 Table — (DOCX) [file ppat.1007484.s016.docx]

| **S3 Table** The information of Clinical samples for clinical data analysis | | | |
| --- | --- | --- | --- |
|  | Clinical samples | |  |
|  | NP(n=15) | NPC(n=55) | p-value |
| Age, years | 40.25 | 48.30 | 0.452^*^ |
| Gender, male | 7(46.67%) | 42(76.36%) | 0.351^﹠^ |
| T stage |  |  |  |
| T1 |  | 15(27.27%) |  |
| T2 |  | 21(38.18%) |  |
| T3 |  | 11(20.00%) |  |
| T4 |  | 8(14.55%) |  |
| N stage |  |  |  |
| N0 |  | 9(16.36%) |  |
| N1 |  | 16(29.09%) |  |
| N2 |  | 21(38.18%) |  |
| N3 |  | 9(16.36%) |  |
| M stage |  |  |  |
| M0 |  | 52(94.55%) |  |
| M1 |  | 3(5.45%) |  |
| M2 |  | 0 |  |
| M3 |  | 0 |  |
| TNM stage |  |  |  |
| I |  | 4(7.27%) |  |
| II |  | 16(29.09%) |  |
| III |  | 21(38.18%) |  |
| IV |  | 14(25.45%) |  |

*Independent t test.  ^&^ Chi-square test.
