# Supplementary material for: EBV-miR-BART1-5P activates AMPK/mTOR/HIF1 pathway via a PTEN independent manner to promote glycolysis and angiogenesis in nasopharyngeal carcinoma
Source: PLoS Pathog. 2018 Dec 17;14(12):e1007484. doi: 10.1371/journal.ppat.1007484 (PMC6312352; doi:10.1371/journal.ppat.1007484)
Supplement: S1 Data — (PDF) [file ppat.1007484.s018.pdf]

Report of Human Cell Line Authentication

Delivery Date: Nov 27th, 2017

Analysis Date: DEC 04th, 2017

Ⅰ. Sample

Sample Name: 'JD2028', labeled as 'HONE1', and was received on Nov 27th, 2017

Ⅱ. Method and Procedure

1. PCR is amplified with STR Multi-Amplification Kit (PowerPlex™16HS System);
2. PCR products are assayed with 3100 DNA Analyzer (Applied Biosystems®).
3. Amplification of gene COX1 and electrophoresis are employed to survey the species of the sample.

Ⅲ. Results

1. The STR profiles of the cell line sample are in the attached table and figure.
2. The search result in ATCC and DSMZ databases.
3. The electrophoresis figure of gene COX1.

HONE1: ①One loci has tri-alleles (D16S539). Contamination of other human cell lines are not found (Figure 1 & Table 1). ②100% matched cell lines are not found in ATCC and DSMZ data banks. (Figure 2 & Figure 3) ③The sample is a human cell line. Contamination of other species cells are not found in the sample (Figure 4).

Operator: Xiaohua Mo

Auditor: Xuanyi Liang

Guangzhou Cellcook Biotech Co., Ltd

GUANGZHOU CELLCOOK BIOTECH CO.,LTD
04 DEC 2017
CELL STR PROFILING
PASS

Figure 1. STR profiles of HONE1 cell line

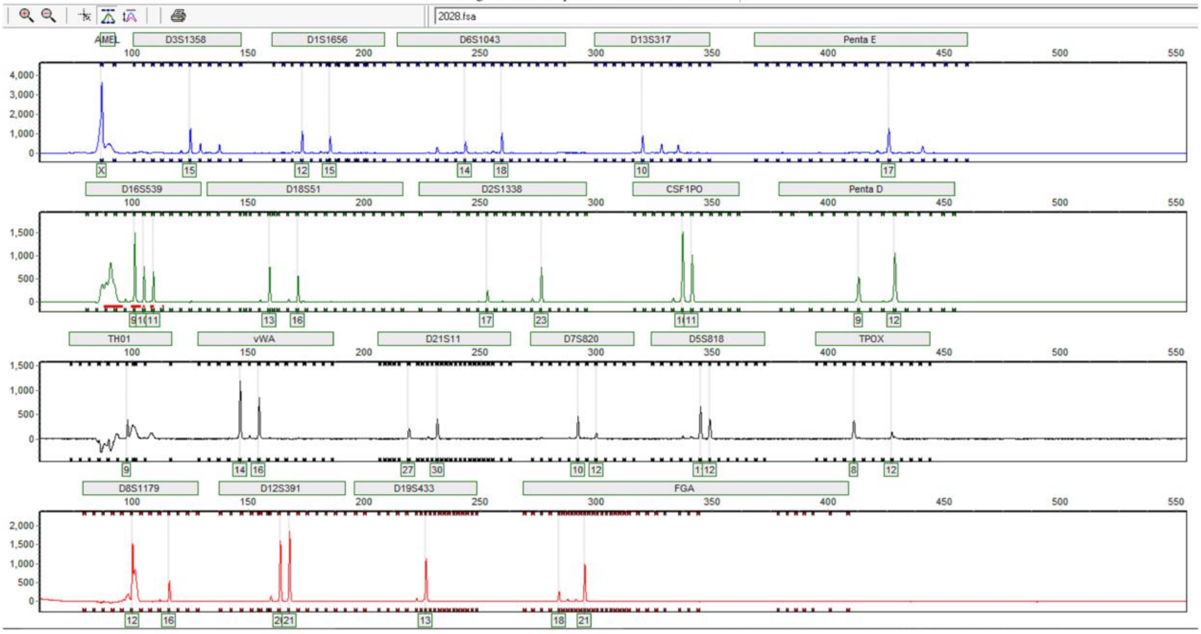

Table 1. STR profiles of HONE1 cell line

	Allele1	Allele2	Allele3
AMEL	x		
D3S1358	15		
D1S1656	12	15	
D6S1043	14	18	
D13S317	10		
Penta E	17		
D16S539	9	10	11
D18S51	13	16	
D2S1338	17	23	
CSF1PO	10	11	
Penta D	9	12	
TH01	9		
vWA	14	16	
D21S11	27	30	
D7S820	10	12	
D5S818	11	12	
TPOX	8	12	
D8S1179	12	16	
D12S391	20	21	
D19S433	13		
FGA	18	21	

Figure 2. Search result in ATCC database

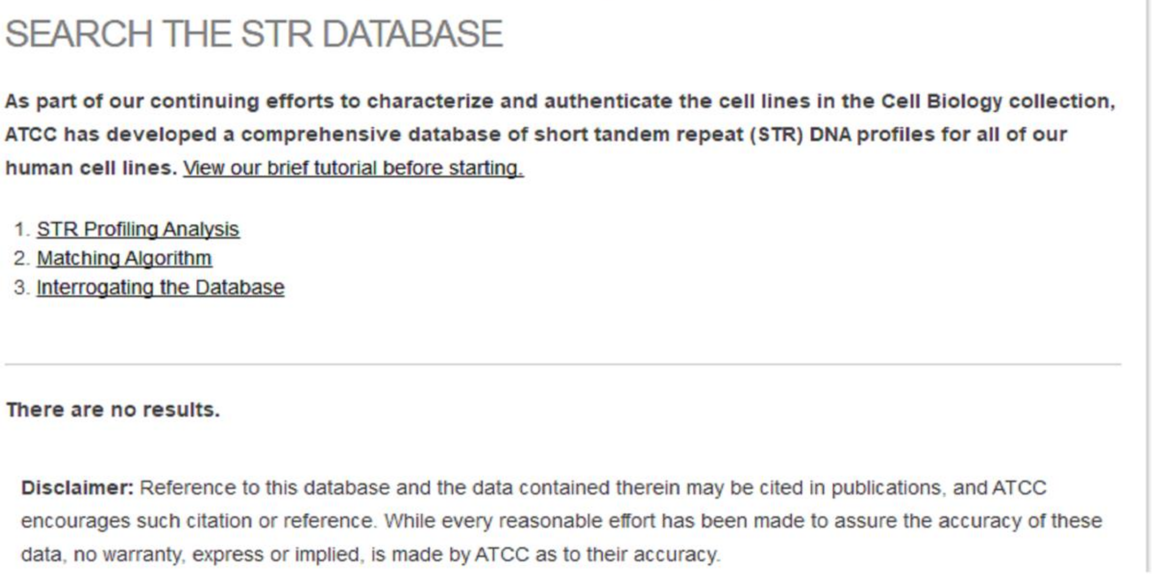
SEARCH THE STR DATABASE

As part of our continuing efforts to characterize and authenticate the cell lines in the Cell Biology collection, ATCC has developed a comprehensive database of short tandem repeat (STR) DNA profiles for all of our human cell lines. View our brief tutorial before starting.

1. STR Profiling Analysis
2. Matching Algorithm
3. Interrogating the Database

There are no results.

Disclaimer: Reference to this database and the data contained therein may be cited in publications, and ATCC encourages such citation or reference. While every reasonable effort has been made to assure the accuracy of these data, no warranty, express or implied, is made by ATCC as to their accuracy.

Figure 3. Search result in DSMZ database

Result of STR matching analysis by your data.

- DSMZ Profile Database -

A graphical presentation is shown at the bottom of this page.

EV	Cell No.	Cell name	Locus names									Figures
			D5S818	D13S317	D7S820	D16S539	VWA	TH01	AM	TPOX	CSF1PO	
	Query (Your Cell)		11, 12	10, 10	10, 12	9, 10, 11	14, 16	9, 9	x, x	8, 12	10, 11	

Figure 4. Authentication of the species of the sample

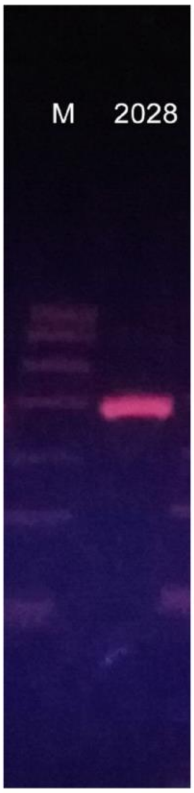

M: Marker. As the size of 700, 600, 500, 400, 300, 200 and 100bp from up to down.

Nine species are checked, as follow: *Homo sapiens* 391bp, *Cricetulus griseus*315bp, *Macaca mulatta*287bp, *Cercopithecus aethiops*222bp, *Rattus norvegicus*196bp, *Canis familiaris*172bp, *Mus musculus*150bp, *Bos Taurus*102bp, IC 70bp

JD2028: The sample. The band size is 391bp which matches the size of human.
